# Multichannel haptic feedback unlocks prosthetic hand dexterity

**DOI:** 10.1038/s41598-022-04953-1

**Published:** 2022-02-11

**Authors:** Moaed A. Abd, Joseph Ingicco, Douglas T. Hutchinson, Emmanuelle Tognoli, Erik D. Engeberg

**Affiliations:** 1 Ocean and Mechanical Engineering Department, Florida Atlantic University, Boca Raton, FL, USA; 2 The Center for Complex Systems & Brain Sciences, Florida Atlantic University, Boca Raton, FL, USA; 3 Department of Orthopaedics, University of Utah, Salt Lake City, UT USA

**Keywords:** Biomedical engineering, Rehabilitation

## Abstract

Loss of tactile sensations is a major roadblock preventing upper limb-absent people from multitasking or using the full dexterity of their prosthetic hands. With current myoelectric prosthetic hands, limb-absent people can only control one grasp function at a time even though modern artificial hands are mechanically capable of individual control of all five digits. In this paper, we investigated whether people could precisely control the grip forces applied to two different objects grasped simultaneously with a dexterous artificial hand. Toward that end, we developed a novel multichannel wearable soft robotic armband to convey artificial sensations of touch to the robotic hand users. Multiple channels of haptic feedback enabled subjects to successfully grasp and transport two objects simultaneously with the dexterous artificial hand without breaking or dropping them, even when their vision of both objects was obstructed. Simultaneous transport of the objects provided a significant time savings to perform the deliveries in comparison to a one-at-a-time approach. This paper demonstrated that subjects were able to integrate multiple channels of haptic feedback into their motor control strategies to perform a complex simultaneous object grasp control task with an artificial limb, which could serve as a paradigm shift in the way prosthetic hands are operated.

## Introduction

The sense of touch is absolutely essential to adroitly control the human hand, and profound deficiencies are observed when tactile sensations are absent or impaired^1,2^. This problem heavily impacts people with upper limb amputations and congenital limb deficiencies^3^. Current prosthetic hands such as the bebionic and i-limb hands have five individually actuated digits^4^, yet only one grasp function can be controlled at a time. Most people commonly use their natural hands to manipulate, grasp, or transport different objects simultaneously. For example, sending commands to multiple fingers while typing on a keyboard, holding a remote control while pressing its buttons, opening a door while holding a bag, or braiding a child’s hair. Such functionalities remain elusive for prosthetic hand users even though new artificial hands are mechanically capable of such feats. Enabling refined dexterous control is a highly complex problem to solve however and continues to be an active area of research because it necessitates not only the interpretation of human grasp control intentions, but also complementary haptic feedback of tactile sensations.

In clinical practice, modern prostheses often use electromyography (EMG) as control signals^5–7^ and amputees typically rely on burdensome visual feedback to monitor the state of the hand^8^. Often, two EMG electrodes are placed over muscles such as the extensor digitorum communis (EDC) and flexor carpi radialis (FCR) on the forearm. Wrist flexor muscles are used to close the hand while the extensor muscles open it. The user must then choose between different grasp types^9^ using a variety of techniques, such as an external button on the bebionic hand or app on a Bluetooth enabled device for the i-limb^10^. Those techniques reduce best practice to manipulation of a single object at once. Also, lack of haptic feedback severely limits the performance of prosthetic hands^11^. This bottleneck in bidirectional information transfer prevents users from fully benefiting from the mechanical dexterity that newer prosthetic hands have to offer.

Due to these and other reasons, recent surveys of people with an upper limb absence have indicated that they would like their prostheses to be dexterous with individual digit control and to have sensory feedback^12–15^. Improvement in these areas could help reduce prosthetic device abandonment rates that are prevalent in populations of limb-absent people around the world, from veterans in the USA^16^, to the general population in Norway^17^, Sweden and the U.K.^13^, among others^18^. In another survey^19^, 74% of respondents stated that they might reconsider prosthesis use if technological upgrades were made at a sensible cost, where people who rejected the use of their prosthetic limb stated that they have more sensory feedback with their residual limb than they do with their artificial limb.

Many researchers have investigated haptic feedback methods for upper limb amputees and people with congenital limb deficiencies^20,21^. Several of the more common minimally-invasive approaches include mechanotactile^20,22,23^, vibrotactile^20,24,25^, and electrotactile feedback^21,26,27^. More recently, soft robotic actuators^28^ have been explored as a noninvasive way to supply haptic feedback. Five soft chambers connected in series and controlled through two pumps and three solenoid valves provided tracking, holding and tapping information^29^. A pneumatic wristband was designed to deliver haptic feedback by inflating eight independent actuators signaling both vibration and pressure^30^. A multi-finger pneumatic actuator system was designed and evaluated to provide the user with haptic sensations^31^. An extendible wristband was built from low-density polyethylene to deliver a combination of directional and force feedback^32^. Furthermore, a soft pneumatic actuator was designed to function as a prosthetic hand socket liner to provide haptic feedback^33^.

**Figure 1:**
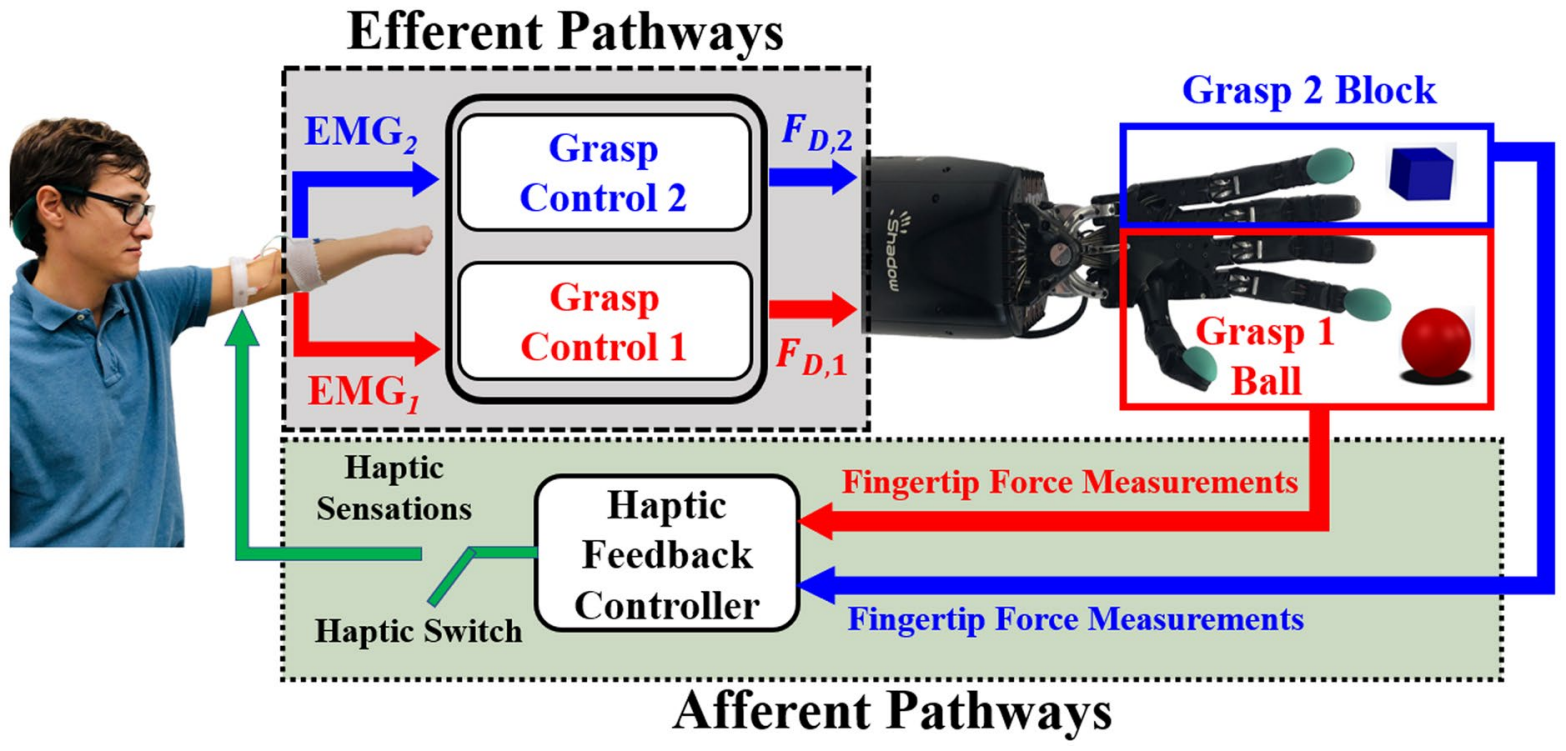
Subjects used two EMG signals (efferent pathways, top) to simultaneously control the distinct grip forces applied to two different objects (top right), in a combinatorial experimental plan with and without haptic (bottom panel) and/or visual feedback. The study demonstrates that multiple channels of bimodal haptic feedback enabled successful grasp and transportation of both objects simultaneously even when vision of the objects was completely occluded [subject’s photograph used with permission].

This paper presents a novel experiment demonstrating that people can integrate multiple channels of haptic feedback into their dexterous artificial hand control strategies to grasp and transport two objects simultaneously, without breaking or dropping them, even when they were unable to see the objects (Fig. 1). This feat of simultaneous EMG control of two different grasp forces was performed by one person with a congenital limb absence and 11 able-bodied test subjects with high success rates even when vision of the artificial hand was completely blocked (Fig. 1). This study takes a step towards to the still elusive goal of dexterous prosthetic limb control.

## Results

The main goal of this paper was to investigate how well people could use multichannel haptic feedback to simultaneously control the grip forces applied to two different objects grasped at the same time with a dexterous artificial manipulator. Furthermore, we explored the role that visual feedback played in this complex multitasking paradigm by systematically blocking visual and haptic feedback in a full factorial experimental design (Fig. 1). Finally, we studied the potential for time savings in a simultaneous object transportation experiment compared to a one-at-a-time approach. To these ends, two sessions of experiments with 12 subjects were conducted over two different days with approximately 90 min to 2 h per session.

### Session 1: grasping and transporting a single object

The first session was designed for the subjects to gain experience with grasping and transporting, without breaking or dropping, a single object at a time with each specific grasp. Breaking an object was conceptualized as a force applied by the robotic hand more than a fixed threshold, an event that was signaled to the subject by vibrotactile actuators embedded in the multichannel bimodal soft robotic armband for haptic feedback. A drop is the escape of the object from the grasp (see details in “Materials and methods” section). The tripod grasp^9^ (thumb opposing index and middle fingers) was used to grip the ball (Movie S1), while the ring and little fingers opposed to the palm were used to grip the block (Movie S2). A haptic guessing game concluded the first session in which subjects were not permitted to view the robotic system while the four possible combinations of objects were placed within the grasp of the hand in pseudo-random order: ball, block, both, neither (Table S4). Participants were asked to determine which object(s), if any, were grasped by the artificial hand using only the multichannel artificial sensations of touch from the custom fabricated soft robotic armband that supplied haptic feedback.

All subjects were able to deliver a single object at a time to the correct location (Fig. S3), and to determine which object(s) were grasped by the hand when vision was occluded using only haptic feedback with high levels of success. Extended results from session 1 are included in the supplementary document.

### Session 2: grasping and transporting two objects simultaneously

**Figure 2:**
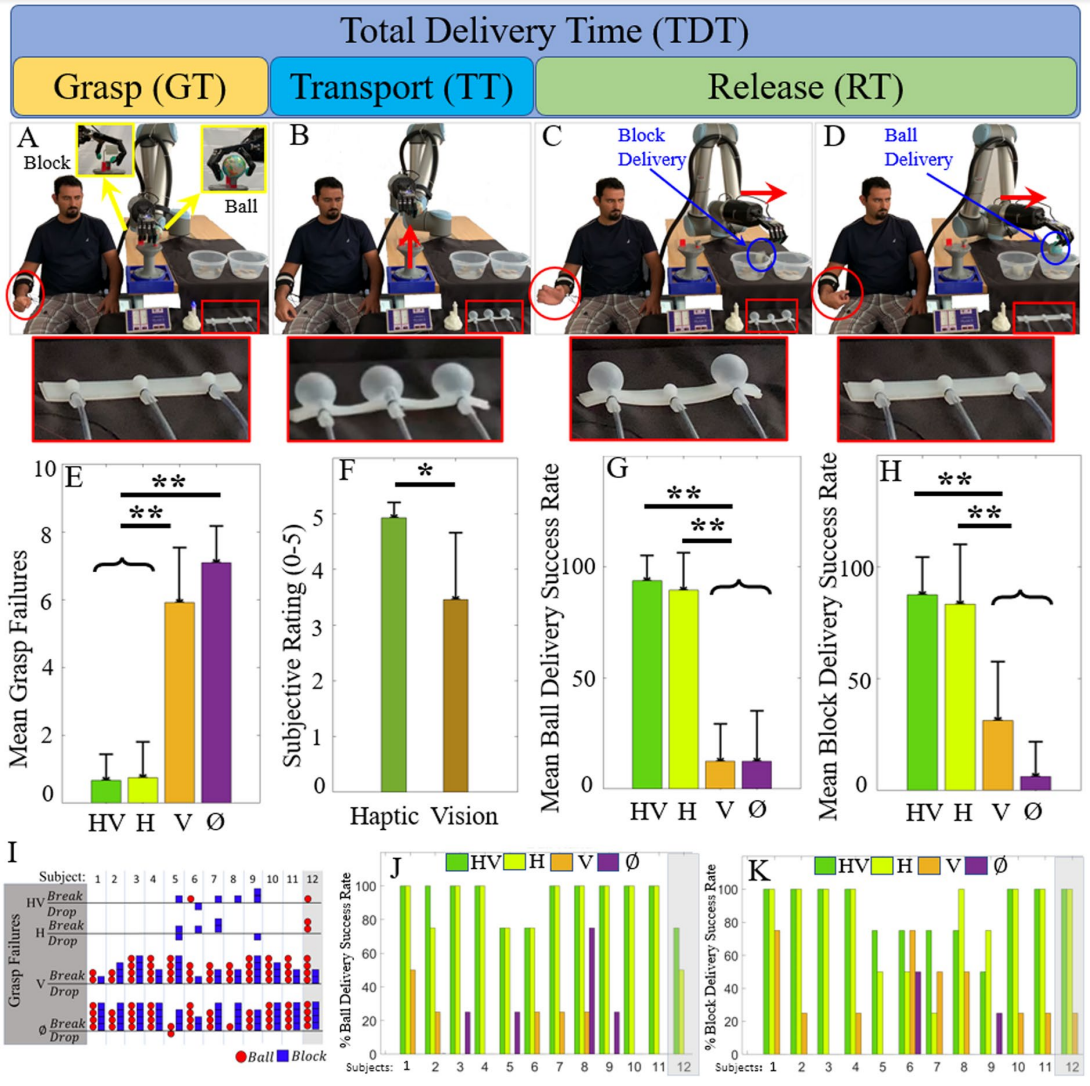
Simultaneous grasp force control overview of success and failure rates. (**A–D**) show consecutive frames of task progression from the simultaneous grasp of both objects (**A**), their concurrent transport (**B**) and deliveries (**C,D**), with an enlarged inset of the soft robotic armband below. Haptic feedback conditions (HV with vision and H without) greatly reduced grasp failure (**E**) when compared to vision alone (V) or no feedback (∅), and increased successful delivery of both objects (**G,H**), leading subjects to report the haptic feedback as more helpful than visual (**F**). Analysis of grasp failure especially showed a more precise use of robotic forces, specifically leading to a striking reduction in object breakage (**I**). Individual subject’s delivery success rates are provided in (**J,K**). The grey highlight indicates the congenital hand-absent subject 12. There was no statistically significant difference among the subjects. *p < 0.05, **p < 0.01.

The second experimental session, which occurred on a subsequent day, involved grasping, transporting and delivering into bins, without breaking or dropping, the same two objects *simultaneously* (Fig. 2A–D, Movie S3). For demonstrative purposes, the soft robotic armband was placed on the tabletop to show how the soft pneumatic actuators inflated proportionally to the forces measured at the tips of the thumb, index, and little fingers (Fig. 2A–D, Movie S4). During experiments with people, this armband was securely wrapped around the upper arm with the inflatable chambers referring haptic information to the subject’s skin. Haptic and visual feedback were systematically disabled or blocked to combinatorically examine the impact that these two variables had on performance of the complex simultaneous control task.

Results from the non-randomized test prior to pseudo-randomization of the independent variables (haptic/visual feedback) are included in the supplemental document (Fig. S6) and followed similar trends as those from the trials where pseudo-randomization of the independent variables were subsequently performed. Data from the pseudo-randomized trials are presented next, which demonstrate the benefits of multichannel haptic feedback.

#### Simultaneous control of grip forces applied to two objects is strongly bolstered by multichannel haptic feedback

Simultaneous grasp force control success rates showed a sizeable benefit from haptic feedback (Fig. 2). The total number of grasp failures was substantially less when haptic feedback was included (HV, for ‘haptic and visual’ feedback; H for ‘haptic’ alone, compared with ‘vision’ alone (V) or ‘no feedback’ (∅)), (Fig. 2E). Success rates for each object and grasp show that haptic feedback substantially improved the subjects’ correct deliveries of both the ball (Fig. 2G)) and block (Fig. 2H); (a successful trial is defined as one in which no failure, drop or break, occurred). The ability to visually monitor the grasping portion of the experiment was altogether much less impactful on the success rates of the subjects, as seen from the lack of significant difference between conditions HV and H (Fig. 2G,H,J,K). When the subjects were given haptic feedback, the overall average success rates were 90.6% ± 14.0% and 86.3% ± 21.7%, with vision (HV) and without vision (H) of the system, respectively. When haptic feedback was disabled, these success rates plummeted to 21.9% ± 21.6%, and 9.4% ± 19.0%, with vision (V) and without visual feedback (∅), respectively.

Importantly for clinical translation, there was no statistically significant difference among the human subjects (p > 0.05), suggesting that all subjects were able to successfully control the system in a comparable fashion, including subject 12 who has a congenital hand absence (Fig. 2J,K).

Analysis of grasp failure type shows that by far, the most common failure was to break an object (Fig. 2I): haptic feedback almost entirely abolished this type of error over the course of this second session. Whether or not visual feedback of the system was available, the effect of haptic feedback was highly significant on both the success and failure rates (p < 0.01), indicating the importance of artificial sensations of touch to successfully perform the complex simultaneous control task. Vision did not significantly impact the success or failure rates (p > 0.05). Interaction among the three independent variables (subjects, haptic, visual feedback) was not significant (p > 0.05).

Finally, subjects qualitatively rated the importance of haptic feedback to be 4.92/5 (98.5% ± 5.5%) on average, while they rated the importance of visual feedback to be 3.46/5 (69.2% ± 24.0%) on average. The U-test indicated that haptic feedback was rated significantly higher than visual feedback (p < 0.05) (Fig. 2F).

#### Time to deliver two objects simultaneously: slower with haptic feedback, but still faster than one at a time

**Figure 3:**
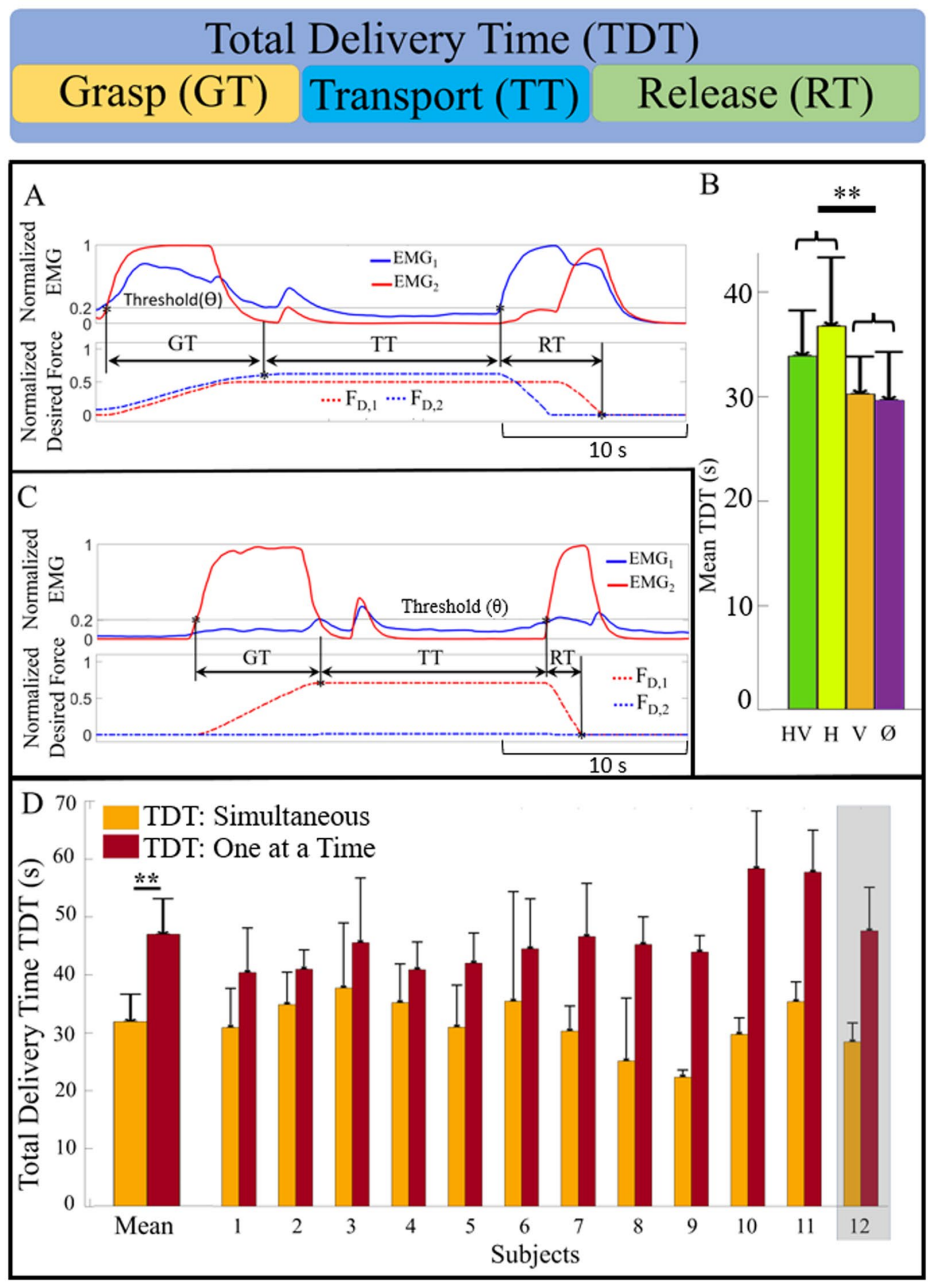
Comparison of total delivery time (TDT) to transport one object at a time versus two objects simultaneously. (**A**) Simultaneous control: The grasp time (GT), transport time (TT), and release time (RT) are illustrated in an exemplary dual-transport trial whose control signals are displayed at the subject level with the envelope of rectified EMG signals and at the robotic level with the desired forces F_D_ used to control the artificial hand. (**B**) Mean TDTs of simultaneous control approach for four feedback conditions with haptic feedback (with Vision ‘HV’; without vision ‘H’) were significantly longer than both conditions without haptic feedback: visual feedback only (V) and neither visual nor haptic feedback (∅). (**C**) For comparison, an exemplary trial shows the control signals for transporting a single object. (**D**) Comparison of the mean TDT for both the single and simultaneous object transportation tasks showed significant improvement with the simultaneous control approach. Each subject, including limb-absent subject 12 demonstrated time efficiency with the simultaneous delivery of both objects. **p < 0.01.

When haptic and visual feedback were available (HV), the total delivery time (TDT, Fig. 3) of two simultaneously transported objects was 32.6 s ± 4.2 s averaged across all 12 subjects. When vision was occluded with haptic feedback enabled (H), the mean TDT increased to 35.4 s ± 6.3 s. When haptic feedback was disabled, the mean TDTs decreased to 29.1 s ± 3.5 s and 28.5 s ± 4.5 s, with (V) and without visual feedback (∅), respectively (Fig. 3B). The TDT was longer with haptic feedback enabled (Fig. 3B), but the faster TDT without haptic feedback came at the expense of significantly more failures (Fig. 2I).

The three factor ANOVA indicated that the subjects and haptic feedback significantly impacted the TDT (p < 0.01) while visual feedback was not significant (p > 0.05). There was also significant interaction between haptic and visual feedback (p < 0.05). However, all other interaction effects were not significant (p > 0.05). See Supplemental Fig. S8 for individual subjects’ TDT data for each feedback condition.

We also compared the TDT for single and simultaneous object transportations. When delivering a single object at a time, we used the sum of TDTs for the ball and block (Fig. S5). This is a conservative estimate of the time needed to transport two objects individually (one at a time), since it does not account for the time required by the robotic arm to return to the starting position to grasp the second object. The sum of TDTs to deliver both objects one at a time was 46.3 s ± 5.6 s when haptic was available and 43.8 s ± 7.3 s when haptic was disabled. Therefore, the mean TDT for delivering both objects, one object at a time, was significantly longer in comparison to their simultaneous delivery for all subjects (p < 0.01, Fig. 3D).

#### Haptic feedback enabled cautious simultaneous grip force control

**Figure 4:**
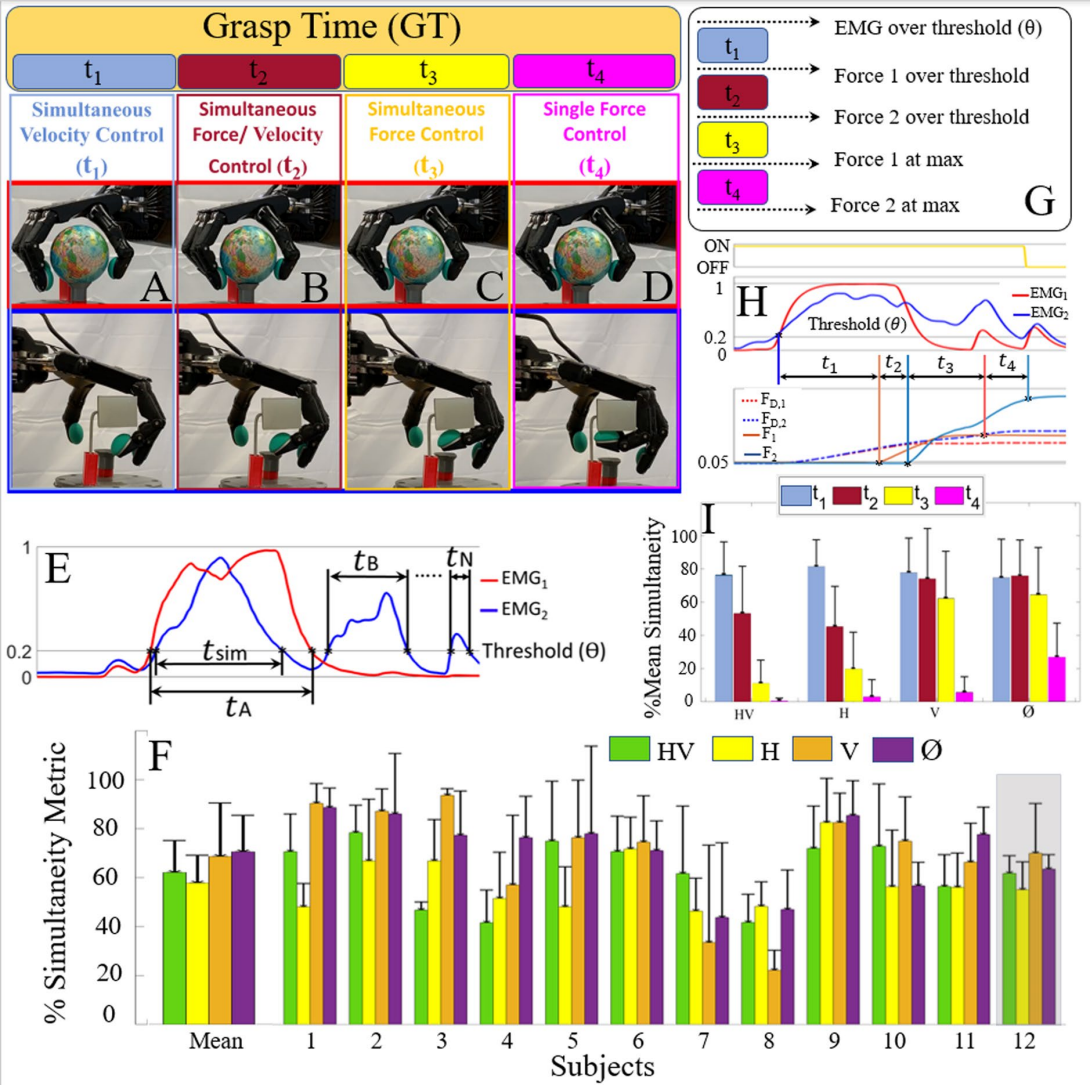
(**A–D**) Comparison of the simultaneity metric under the four time windows: t_1_, t_2_, t_3_, and t_4_. (**E**) Illustrative data showing instances where both grip forces were simultaneously or individually controlled for fine grip force adjustments during the grasp phase of the experiments, prior to transportation. (**F**) Percentage of time that both grip forces were simultaneously controlled by all 12 subjects during the entire grasp phase. The gray highlight indicates the limb-absent subject 12. (**G**) Table and (**H**) sample data of efferent and afferent events that separate the four key time periods during the simultaneous control task. (**I**) Overall averages of all 12 subjects during timeframes t_1_–t_4_ with a comparison of the simultaneity metric under the four possible combinations of independent variables: {1} both haptic and visual feedback (HV), {2} only haptic feedback (H), {3} only visual feedback (V), and {4} neither haptic nor visual feedback (*ϕ*).

To investigate the extent to which haptic and visual feedback affected simultaneous motor control during the grasp phase of the simultaneous control experiments (Fig. 4A–D), we defined a metric of simultaneity as the percentage of time with simultaneous control:

1 $${\text{Simultaneity}} = \frac{{t_{sim} }}{{t_{A} + t_{B} + \cdots + t_{N} }}.$$

Here, t_A_, t_B_, ……, t_N_ indicate all durations of time where only a single EMG grasp control signal was above the minimum activation threshold (ϴ) and t_sim_ is the amount of time where both EMG signals were simultaneously above the same activation threshold (Fig. 4E). The simultaneity metric was calculated only on the grasp portion of the task (Fig. 4A–D) since this is the stage of the task where subjects were trained to control their EMG signals simultaneously due to the design of the distinctly different delivery locations of the two objects (Fig. 2C,D).

When subjects had both haptic and visual feedback (HV), they controlled their desired grip forces on both objects simultaneously 61.6% ± 12.9% of the time, on average. When haptic feedback was available, but vision was occluded (H), the simultaneity metric decreased to 57.3% ± 11.3%, on average. However, the simultaneity metric increased when haptic feedback was disabled to 68.1% ± 21.6% and 70.0% ± 14.8% with (V) and without visual feedback (∅), respectively (Fig. 4F).

The three factor ANOVA showed that there was no significant difference between the subjects (p > 0.05); however, both haptic and visual feedback significantly impacted the simultaneity metric (1), (p < 0.01). The interaction between haptic and visual feedback variables was also significant (p < 0.01). All other interactions among independent variables were not significant (p > 0.05).

We further subdivided the simultaneous grasp task into four time intervals (t_1_ to t_4_) corresponding to the onset of crucial afferent and efferent events distinguishing simultaneous/split velocity and force control. Briefly, we defined t_1_ as being the simultaneous velocity control portion (Fig. 4A)—the interval between engagement of EMG activity and the onset of one supra-threshold robotic fingertip force (whichever grasped object was contacted first). The next segment of time was simultaneous force/velocity control, t_2_, when one grasp was still closing and had not yet contacted the object while the other grip was in contact with the other object (Fig. 4B). This time segment lasted until the grip forces on both objects were over a minimal threshold, ensuring that both objects were contacted by the hand. Time segment t_3_ was the simultaneous grip force control portion (Fig. 4C) which lasted until one of the fingertip forces reached its maximum (typically a stable level preserved through grasp and transportation). Finally, t_4_ was the segment of time when control of a single grip force was required, from the end of t_3_ until when a maximal force was applied to the second object (Fig. 4G). Note that we used fingertip forces rather than the corresponding pressures in the soft robotic armband to facilitate the quantitative comparison of conditions with and without haptic feedback.

A detailed analysis of the four intervals of grasping behavior (t_1_ to t_4_, (Fig. 4H)), revealed that neither haptic nor visual feedback significantly affected t_1_ (onset of efferent to onset of afferent), (Fig. 4I). In contrast, t_2_ and t_3_ revealed large differences between the two conditions with haptic feedback (HV, H), and the two conditions without (V, ∅). In particular, haptic-enabled conditions exhibited less simultaneity during the t_3_ time period (simultaneous force control, Fig. 4C), where a more cautious approach was taken to avoid breaking either object. As expected, simultaneity was lowest during time segment, t_4_ under all four feedback conditions but showed a marked increase in the no feedback case (∅), corresponding to increased grasp break failures (Fig. 2I). See also Supplemental Fig. S9 for all subjects’ simultaneity scores for each of the four different time periods.

## Discussion

### Multiple channels of haptic feedback enabled simultaneous proportional control of grip forces applied to two objects

This paper has demonstrated that people were able to integrate multiple channels of haptic feedback into their EMG motor control strategies to grasp and transport two objects simultaneously, without breaking or dropping them, even when vision of the objects was occluded. Results showed that the multichannel bimodal haptic feedback from the soft robotic armband enabled high success rates with the congenital limb-absent subject and the 11 subjects without upper limb deficiencies. Furthermore, the simultaneous control approach was shown to improve the time required to transport and deliver both objects in comparison to a one-at-a-time approach commonly used in prior works. Important for clinical translation, there were no significant differences between the limb-absent subject and the other subjects for the key performance metrics in the tasks.

While it is commonly known that haptic feedback can effectively substitute and augment visual feedback during force control with an artificial hand^25^, this paper provides new information showing that people can successfully integrate multiple channels of bimodal haptic feedback to proportionally control the forces applied to two different objects grasped simultaneously. After a brief but well-structured training protocol performed with subjects who were naive to EMG prosthesis control, we found signs of a cognitive mapping between robotic fingertip touch percepts from the tactile sensors and their referred sensations via the haptic interface worn on the subjects’ arms. Specifically, subjects nearly eliminated all grasp failures for both objects (Fig. 2E,I) and greatly increased the number of successful deliveries during the simultaneous control experiments (Fig. 2G,H,J,K). The comparison of success/failure rates with and without haptic feedback was highly significant (p < 0.01), while visual feedback did not significantly impact the success/failure rates (p > 0.05). This was also true for the non-randomized sequence of simultaneous grasp transportation experiments (Fig. S6), which was performed just prior to the pseudo-randomized experiments. Furthermore, subjects qualitatively rated haptic feedback as significantly more important than visual feedback even when vision was available (Fig. 2F) because there was often little to no visually perceptible warning before grasped objects were broken or dropped. These reasons are the most likely explanation for the lack of significant interaction between the haptic and visual feedback variables on the success/failure rates. Our behavioral performance data demonstrated that for the success/failure rates associated with dual-object manipulation (Figs. 2, 3, 4), hapsis was dominant and vision only secondary. Nevertheless, we suggest that their multisensory integration will generally be preferable in the tasks of daily life^34^.

### Haptic feedback slowed but refined simultaneous task performance

Absence of haptic feedback yielded faster delivery times (Fig. 3B), at the expense of significantly more grasp failures (Fig. 2I). The faster delivery of objects without haptic information is likely due to subjects simultaneously controlling the grip forces on both objects a higher percentage of the time (Fig. 4F) in a heavy-handed manner without any inhibitory feedback cues. Therefore, it was not necessarily a desirable aspect of simultaneous task performance; rather, it reflected a poor speed-accuracy tradeoff in this case, where objects were broken, and artificial hand components were overexerted. Furthermore, haptic feedback was also found to increase the TDT in the single-object transportation experiments during Session 1 (Fig. S5). It is important to note that non-excessive grip forces enabled by haptic information promise to spare prosthetic hands from premature mechanical stress, aging and failure^35^, with the benefit of longer usability and lower cumulative cost for the patients.

An interesting finding concerns the subtle interplay between haptic and visual feedback, which interacted significantly based on the simultaneity metric (1), (Fig. 4) and the TDT (Fig. 3) but did not interact on the success or failure rates during the complex simultaneous control tasks (Fig. 2). This interaction is likely due to the subjects’ varying use of those two feedback modalities along the four time periods t_1_ to t_4_ (Fig. 4I, Fig. S9). At the beginning of each grasp cycle (t_1_), subjects adopted a strategy in which they simultaneously increased the desired grip forces. Because the fingertips had not touched either object during t_1_ (Fig. 4A), velocity control was solely achieved from visual feedback, when available. The faster TDT in the HV case compared to the H case is likely because the subjects were able to visually see when the fingers were about to touch the objects, allowing them to more quickly close the hand without fear of breaking either object accidentally. In the later stages of grip force control, haptic feedback enabled subjects to be more nuanced in their control to avoid breaking an object. From the onset of t_2_ through t_3_, some subjects would fine-tune the grip forces one at a time to precisely dial in the fingertip force levels as desired to successfully implement this complex task. This fine-tuning during t_3_ explained the less prevalent use of simultaneous control and a slower TDT when haptic information was present. It contrasted starkly with the situation where neither haptic nor visual feedback were enabled (**∅**): both EMG signals were co-activated more often, resulting in breaking both objects (Fig. S7) significantly more along with a faster TDT. Reduced simultaneity of EMG during t_3_ was not surprising since there were times during t_3_ when one grip force was satisfactory while the other force was too low, meaning that simultaneous EMG co-activation during t_3_ was not always necessary. This set of findings suggests that haptic and/or visual cues can serve inhibitory roles in the complex simultaneous control task and bolster overall success rates by preventing break and drop failures, as is patently obvious from Fig. 2I. In summary, the higher scores of simultaneity when haptic information was absent (Fig. 4I) reflect the inability to sense the grip force levels for both objects, and this occurred at the cost of object breakage (Fig. 2I). Conversely, when haptic information was present, EMG co-activation was more intermittent and lowered the overall simultaneity metric, but the nuanced adjustments led to better quality of motor control. The highly significant impact of multichannel haptic feedback clearly demonstrated that people used their awareness of the forces simultaneously applied to the two grasped objects to precisely grasp and transport them, even when vision was occluded.

### Simultaneous control enabled significant time savings during object transportation

On average, all 12 subjects saved significant amounts of time when delivering two objects simultaneously instead of one at a time (Fig. 3D) with negligible impact on the task success and failure rates (Fig. 2, Fig. S3). It is also important to mention that this task was designed to transport two objects a short distance across a table, yet the simultaneous transport approach yielded a striking improvement in overall task completion time even though the TDT metric did not account for the arm’s return time to grasp the other object during the single object transportation experiments. This increase in efficiency would become even more pronounced in many other common situations involving a greater transportation distance, such as carrying two objects from a desk to a vehicle, for example.

### Future applications of simultaneous grip force control

Results showed that all subjects had high success rates while grasping and transporting two objects simultaneously without breaking or dropping them, even when they were not able to see the two objects. None of the subjects had significant prior use of EMG-controlled artificial hands, yet they were able to learn to harness this multitasking functionality after two short training sessions. As such, the contributions of this paper could catalyze a paradigm shift in the way current and future artificial hands are controlled by limb-absent people. While we have demonstrated potential to simultaneously control two specific grasp types (Fig. 2A–D), the next obvious question is how a person could select which grasp types to use; there are several possibilities in the present and future. In the present, an app such as the commercially available i-limb iPhone app or button on the bebionic hand^4^ that currently allows users to select the grasp function of the hand could be readily modified to allow the user to select any number of functions for simultaneous control, such as those demonstrated in this paper (Fig. 2A–D), which are well within the capabilities of several commercially available prosthetic hands. But as prosthetic hand sophistication progresses with devices such as the modular prosthetic limb^36^, Hannes hand^37^, Mia hand^38^, and Deka Hand^39^ pointing toward increasing functionality, more seamless integration within daily life could be realized via EMG pattern recognition techniques to detect the user’s desire to simultaneously control multiple functions^40–48^. In the supplemental material, we demonstrate simultaneous and proportional control of a card being pinched between the index and middle fingers at the same time that the thumb and little finger were used to unscrew the lid of a water bottle (using the approach described in Ref.^49^, see Fig. S11, Movie S5). Another example we demonstrate shows a ball that was grasped with a tripod grip while the little finger was simultaneously used to toggle a light switch (Fig. S10, Movie S6). Finally, the information discovered in this paper could also be used in the future frameworks of highly complex bimanual operations^50,51^, such as those required of surgeons^52^ and guitarists^53^, with the goal of enabling upper limb-absent people to pursue vocations currently unattainable to them.

While the focus of this paper has been on high-level human capability for multichannel sensorimotor integration, autonomous low-level approaches could improve the performance of this simultaneous control strategy in daily life. For example, the weight of each individual object could be largely unknown to the limb-absent subject who would likely only be cognizant of the combined weight of both objects after lifting them. This might lead to suboptimal grip forces applied to the objects, which could be ameliorated via a soft-synergy approach with mass-dependent variable gains^54^, or through a grasped object slip prevention algorithm^55^. Additionally, techniques to automate the shoulder, elbow, and wrist joints could be highly beneficial to reduce the cognitive burden on the amputee to multitask by sensing upper body compensatory motions^56^ or shoulder kinematics with respect to the grasped object locations^57^ to autonomously plan smooth prosthetic arm trajectories. These types of motion planning algorithms could reduce unnecessary oscillations in grip forces and corresponding haptic sensations during object transportation^58^ while reflexive compensation for inertial loads during transport could be used to proactively prevent slip of the grasped objects^59^.

This study, developed within a laboratory setting, could gain from being extended to more realistic situations that do not only involve repeatedly transporting the same two objects. Since this paper is the first (to our best knowledge) to demonstrate the feasibility of this complex simultaneous control task while integrating multiple channels of haptic feedback noninvasively, further experiments are warranted with wearable prosthetic limbs in unstructured tasks of daily life. We posit that in the future, more research on the impact that multiple channels of haptic feedback has on the ability to multitask with an artificial hand will be an important question to answer so that limb-absent people can exploit the full dexterity of next-generation prosthetic limbs^60–62^, with which there seem to be stiff barriers to dexterous manipulation of multiple objects in complex tasks of daily life. We argue that this paradigm shift will ultimately serve well the users of prosthetic hands who have long awaited advances in dexterity.

## Materials and methods

### Human subjects

Twelve human subjects participated in these experiments (six female). One male subject had a congenital hand absence, and the remaining eleven human subjects had no amputation or congenital limb deficiencies. All participants gave informed written consent under a protocol approved by Florida Atlantic University’s IRB, which was in accordance with the declaration of Helsinki. The participants depicted in the figures gave informed consent for their images to be published.

### EMG algorithm for simultaneous control

**Figure 5:**
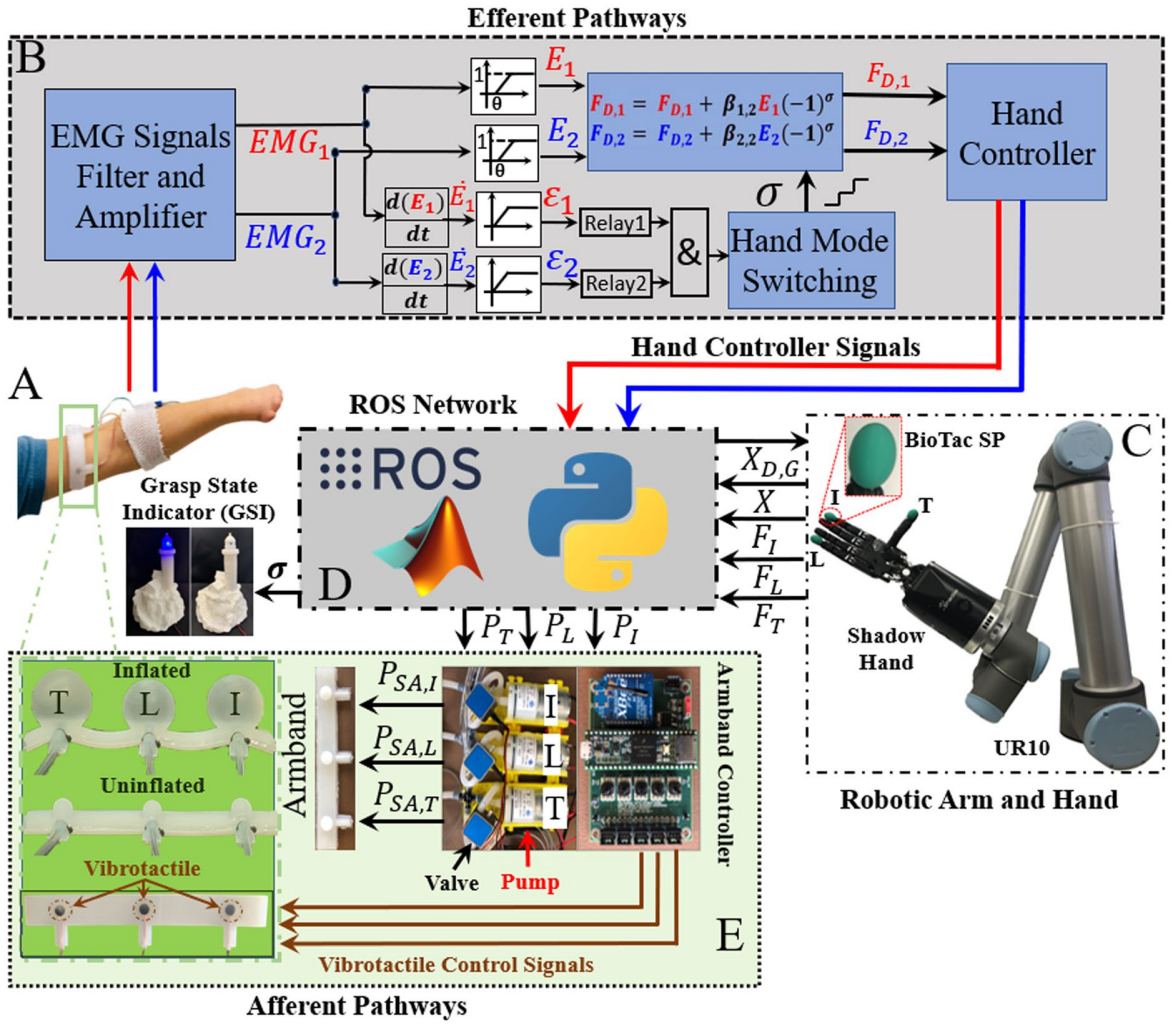
System Configuration: all the system components are interacting with each other through ROS, Python, and MATLAB/Simulink. (**A**) Two EMG surface electrodes were placed on the forearm of the human subject to record forearm muscle activities. The soft robotic armband was located on the upper arm. (**B**) EMG signal processing (Efferent Pathways) was done in Simulink to control the (**C**) Dexterous Shadow Hand and UR10 Robot arm that were interfaced via (**D**) ROS. (**E**) The soft robotic armband had a pump, valve, and pressure sensor for each of the three soft actuators. Vibrotactile actuators were also co-located with the soft actuators to give bimodal multichannel haptic feedback for both objects grasped simultaneously (Afferent Pathways).

The 12 subjects controlled a Dexterous Shadow Hand (Shadow Robot Company, London, U.K.) with two EMG surface electrodes that were placed on their forearms. Activity from the FCR muscle (EMG_1_ to control tripod grasp 1 of the ball) and EDC muscle (EMG_2_ to control the ring/little fingers—grasp 2 of the block) (Fig. 5) were filtered, rectified and amplified with Myolab II (Motion Control, Inc., Salt Lake City, USA). Next, the two EMG signals for grasp G ∈ 1, 2 were normalized on a scale of 0 to 1 and a subject-specific lower threshold (θ) was set to remove baseline noise from the EMG:

2 $$E_{G} = \left\{ {\begin{array}{*{20}l} 0 \hfill & {if\,|EMG_{G} |\, < \theta } \hfill \\ {\beta_{G,1} |EMG_{G} |} \hfill & {if\,|EMG_{G} |\, \ge \theta } \hfill \\ \end{array} } \right\}.$$

*β*_*G*,1_ are constant gains for each EMG control signal for the two different grasps, and E_G_ is the processed EMG. Within a hybrid force–velocity control scheme, subjects could control the velocity or force of either grip simultaneously or individually. After contacting an object, the subjects could independently or simultaneously increase or decrease the desired grip forces *F*_*D,G*_ which were maintained by the force control loops:

3 $$F_{D,G} = F_{D,G} + \beta_{G,2} E_{G} ( - 1)^{\sigma } .$$

*β*_*G*,2_ are gains for each grip force controller. Thus, subjects could increase the desired forces *F*_*D,G*_ for either grasp controller simultaneously or independently or could decrease the desired forces (simultaneously or independently) in a proportional manner by co-contracting muscles at different relative levels. Switching the opening/closing mode of the hand was enabled via a rapid co-contraction to increment σ, which is defined as:

4 $$\sigma = \left\{ {\begin{array}{*{20}l} {\sigma{+}{+}} \hfill &\quad {if\,\varepsilon_{1} \,{\text{and}}\,\varepsilon_{2} > \alpha } \hfill \\ \sigma \hfill & \quad{{\text{else}}} \hfill \\ \end{array} } \right\}.$$

ε_1_ and ε_2_ are the positive portions of the derivatives of the two EMG signals (2), and α is a subject-specific threshold that was determined empirically during the initial portion of experiments. This approach allowed only a single pulse of a rapid muscle contraction to pass into relay blocks. The relay blocks converted the biocontrol signals into clean on–off indicators of rapid muscle co-contractions which were used to distinguish between simultaneous control of both grip forces and the desire to switch the operational mode of the hand between opening or closing. A switching signal logic block was used to increment (σ) so that the user could comfortably increase or decrease desired forces (3) simultaneously or independently (Fig. 5B). A rapid co-contraction toggled the open/close mode of the hand. To help subjects remain aware of the open/close mode of the hand, a grasp state indicator (GSI) was placed in view to indicate the open/close mode of the hand (Fig. 5).

### Robotic system configuration

The dexterous E3M Shadow Hand has 24 tendon driven joints^59^, affording dexterity comparable to the human hand. The Shadow Hand was mounted onto a UR10 robotic arm (Universal Robot, Odense, Denmark) to enable a well-controlled method to transport grasped objects. Three BioTac SP sensors (SynTouch Inc, CA, USA)^63^ were mounted onto the thumb (T), index (I), and little (L) fingers of the hand to measure the fingertip forces applied to the grasped objects (Fig. 5C). The haptic feedback was delivered via a custom fabricated bimodal soft robotic armband^64^ that was placed on each subject’s arm (Fig. 5A,E). There were three nonlinear air pressure controllers, one for each of the three soft robotic actuators. The Arduino IDE was used to implement the controllers for the soft actuators with a Teensy 3.6 (Fig. 5E, right). Each controller used a pump, valve, and pressure sensor (Fig. 5E, center) to map the forces at each of the three fingertips (Fig. 5C) to air pressures proportionally pumped into the three soft actuators on the wearable robotic armband (Fig. 5A,E). Co-located vibrotactile stimulators (Fig. 5E) were activated to indicate if one or both grasped objects were broken by exceeding predetermined grip force thresholds.

**Figure 6:**
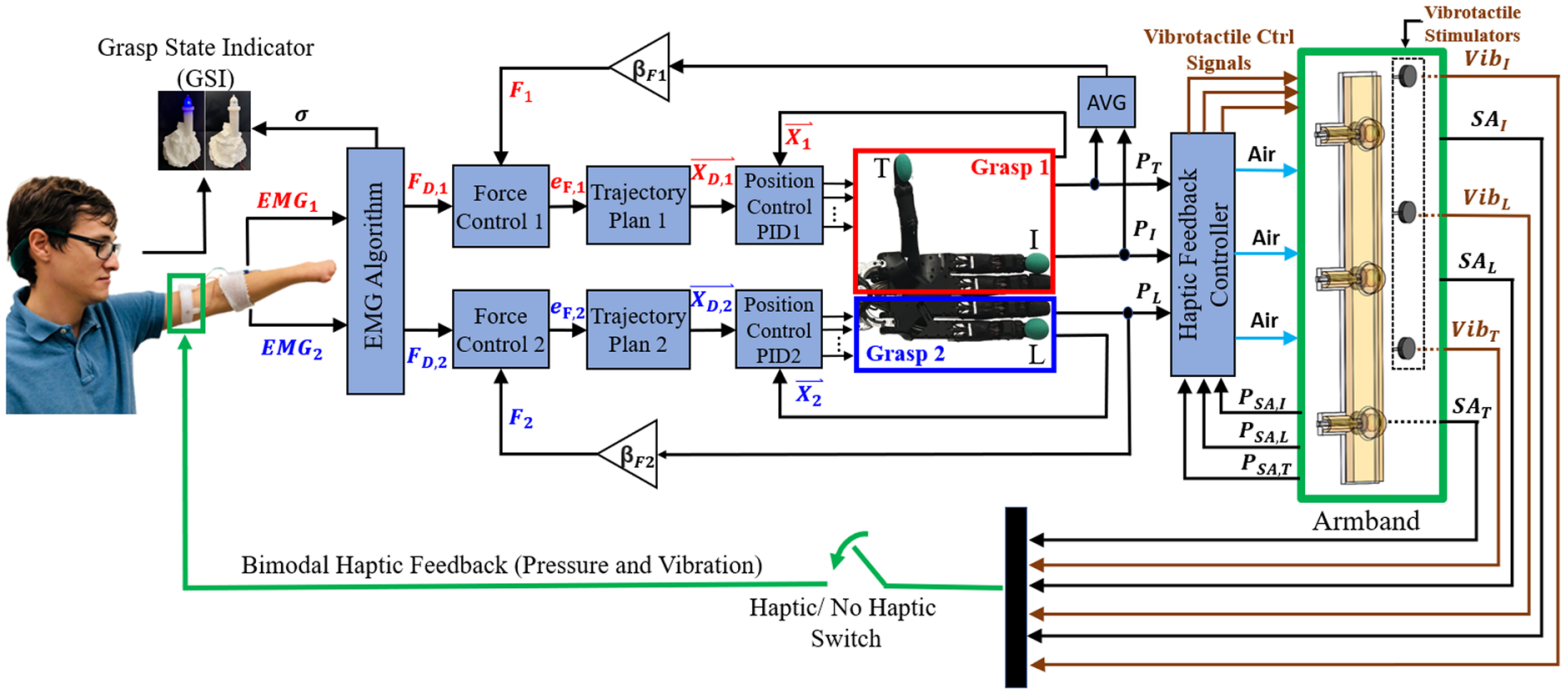
Control system overview. Hybrid force–velocity controllers to simultaneously control the forces applied to two different objects grasped by a dexterous Shadow Hand. The haptic feedback controllers (right) provided bimodal multichannel tactile information from three of the robotic fingertips, using the soft actuators and fast-acting vibrotactile information, used here to signal breakage of one or both objects.

The analog EMG signals (Fig. 5A) were digitized by another Teensy 3.6 board and were interfaced with MATLAB/Simulink through the Robot Operating System (ROS) toolbox (MathWorks, Inc., Natick, USA). All the components communicated with each other through the ROS environment (Fig. 5D)^65^. Simulink was used to design the human subject training protocols and the two hybrid force–velocity controllers^66^ that enabled simultaneous control of velocities and grip forces applied to the two objects grasped by the hand (Fig. 6).

### Grasp synergy trajectory plans

To avoid overwhelming the human user, the Shadow Hand was programmed using grasp synergies^67^, whereby a sizable joint space was mapped into a smaller biocontrol space^68^, effectively limiting hand control to two DOFs in this paper, one for each grasp and object (Fig. 1). In this case, EMG_1_ was used to control the tripod grasp for the ball and EMG_2_ was used to control the ring and little fingers to grasp the block. Quintic polynomials were planned for the joint angle trajectories to provide smooth motion profiles (Fig. S1). All joint angle trajectories were normalized over the same 0–1 scale as the EMG signals (2) so that all joints in each of the two grasps were temporally synchronized to the corresponding EMG biocontrol signal (Fig. S1). Note, however, that the technique is scalable to any number of grasps or functions (Movies S5, S6), depending upon the capabilities of the artificial hand, for which there are control inputs, such as grasping, catching^67^ or unscrewing different objects^49^.

### Bimodal soft robotic armband for multichannel haptic feedback

To provide haptic feedback, a custom fabricated multichannel bimodal soft robotic armband was designed with soft actuators to convey a proportional sense of contact forces; vibrotactile stimulators were included to indicate if the grasped object(s) had been broken. Prior research has shown that spatial discrimination of five sites with mechanotactile feedback was difficult for some participants^20^. Accordingly, the novel soft robotic armband in this paper was designed for haptic feedback at three locations corresponding to the thumb, index, and little finger, a sufficient number to convey the amplitudes of the forces applied to both objects grasped by the hand. The armband has three air chambers, each of which proportionally corresponds to one of the three BioTacs equipped on the Shadow Hand fingertips. The armband is also equipped with three co-located vibrotactile actuators that would vibrate to alert the subject if the object(s) in the grasp(s) had been broken (if one or both force thresholds was/were exceeded). Thresholds for breaking the objects were chosen to provide a moderate challenge for the subjects to grasp and transport the objects without breaking or dropping them.

### Manufacturing the soft robotic armband

**Figure 7:**
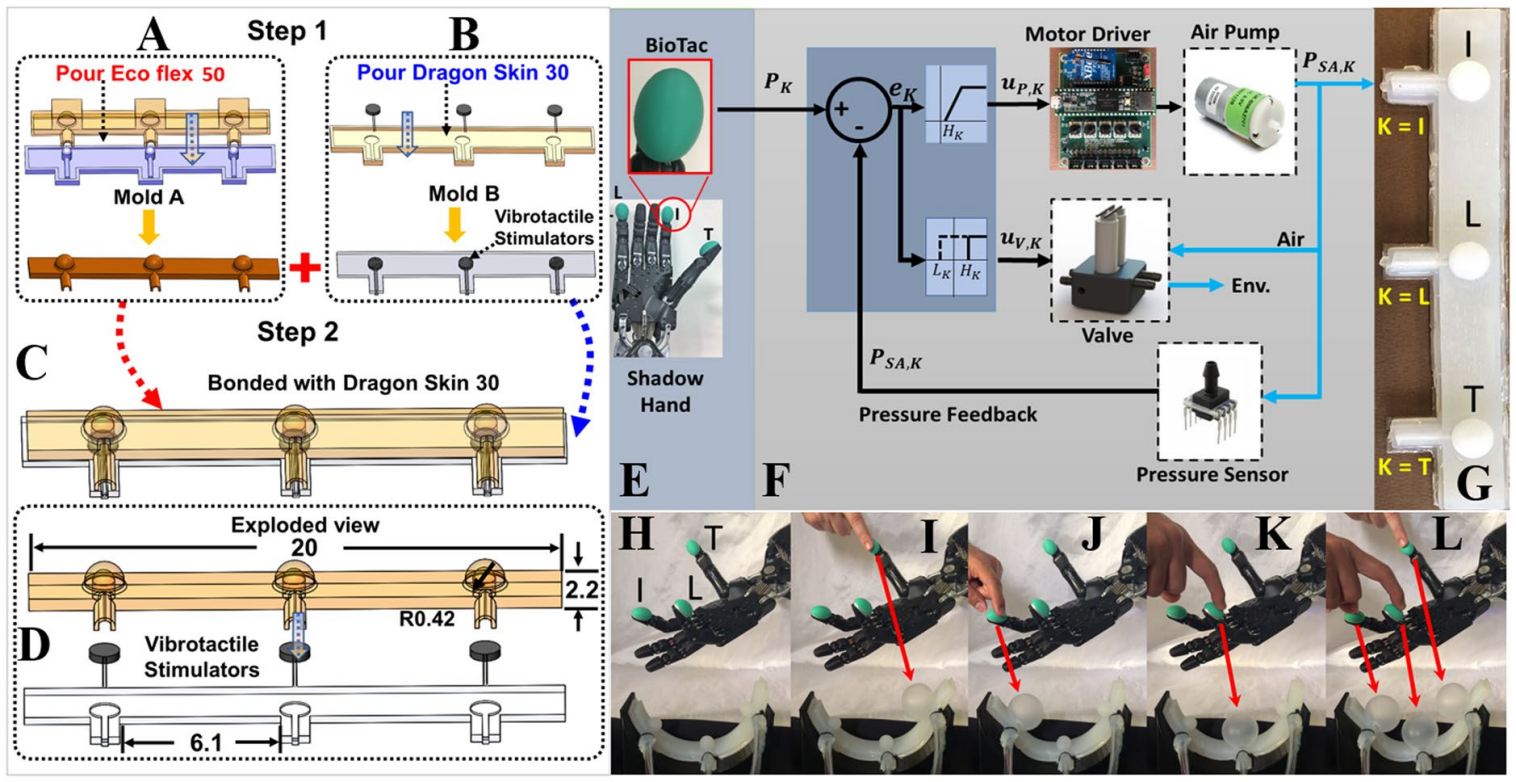
Soft robotic armband: manufacturing and control. (**A**) The upper and foundation molds are mated to manufacture the actuation part of the armband. (**B**) The vibrotactile actuators are embedded in the base. (**C**) The actuation and the base parts are bonded together. (**D**) Exploded view showing the armband components, units of cm. (**E**) Shadow Hand and BioTac SPs. (**F**) Soft actuator nonlinear pressure controllers. (**G**) Soft robotic armband where T, L, and I indicate the thumb, little and index fingers, respectively. (**H**) Three BioTac SPs attached to the Shadow Hand are shown at rest. Note the deflated state of all three air chambers on the armband (placed below the hand for illustrative purposes). (**I**) Applying pressure to the thumb (**J**) index, and (**K**) little fingertips individually or (**L**) simultaneously resulted in proportionally scaled pressures within the soft robotic armband actuators, implemented by each of the three nonlinear controllers.

Three molds were 3D printed to manufacture the armband: the foundation, insertion, and base molds (Fig. 7A) to create the actuation and base layers of the armband. The foundation and the insert molds were mated to create the cavity for the top layer. The armband layers were cast independently (Fig. 7B,C) and then were bonded together to create the armband structure (Fig. 7D). Armband layers were molded from ISO 10993-10 certified skin-safe materials of different Shore hardness. The top layer was molded from EcoFlex-50 (Smooth-On, PA, USA), while the base layer was molded from a more rigid material (Dragon-Skin 30 (Smooth-On)), to direct the soft actuator expansion towards the user’s skin when inflated. Vibrotactile stimulators were inserted into the base mold (Fig. 7B) to convey high-frequency tactile sensations alerting the subjects if one or both of the grasped objects had been broken. For more details regarding the design and manufacturing the soft robotic armband, see^64^.

### Nonlinear pressure controllers for soft actuators

Nonlinear pressure controllers were developed (Fig. 7E,F) so that the air pressures within soft actuator K (K ∈ I, L, T) matched the pressures measured by the corresponding BioTac fingertip sensors. Each pressure controller was designed to control a pump and a valve (Fig. 5E, center) for each air chamber in the soft robotic armband to minimize the error:

5 $$e_{K} = P_{K} - P_{SA,K} .$$

P_K_ is the robotic fingertip pressure measured by BioTac sensor K and P_SA,K_ is the pressure within the corresponding soft robotic actuator K (Fig. 7E,F). The error for each haptic controller K was split into two branches: one for inflation and another for deflation. For inflation, each nonlinear controller K activated a pump to inflate the soft robotic air chamber K with the pump control signal:

6 $$u_{P,K} = \left\{ {\begin{array}{*{20}l} {sat(\beta_{K} e_{K} )} \hfill & {if\,e_{K} \, > \,H_{K} } \hfill \\ 0 \hfill & {if\,e_{K} \, \le \,H_{K} } \hfill \\ \end{array} } \right\}.$$

*β*_*K*_ was a constant gain for each soft actuator controller K. *H*_*K*_ is the upper limit of the deadband used to eliminate chatter. To aggressively minimize the error, a saturation function was used to enable high gains and rapid system response.

A normally open 12 V 2-way valve (TCS Micropumps Ltd) was utilized to control the airflow direction, either into the armband air chamber for inflation or into the atmosphere for deflation. The solenoid valve control signal (*u*_*v,K*_) was either on or off to close or open the valve, respectively:

7 $$u_{v,K} = \left\{ {\begin{array}{*{20}c} {Close} & {if\,e_{K} \, > \,H_{K} } \\ {Open} & {if\,e_{K} \, \le L_{K} } \\ \end{array} } \right\}.$$

*L*_*K*_ is the lower threshold of the error deadband to minimize valve chatter and enable the stable maintenance of a constant pressure within soft robotic actuator K, and *H*_*K*_ > 0 > *L*_*K*_. Demonstration of this function can be seen when a person sequentially squeezed the three BioTac sensors on the fingertips of the Shadow Hand: the three soft robotic actuators correspondingly inflated (Fig. 7H–L) (Movie S4).

### Vibrotactile sensory feedback

Three vibrotactile stimulators were integrated into the bimodal soft robotic armband to rapidly deliver urgent information about the grasped object(s), such as a rapid deformation indicative of a broken object. This is similar to how rapidly adapting mechanoreceptors respond to high frequency touch sensations^1^. For example, if a person cracked a brittle object, he or she would perceive rapid fluctuations in force. Three vibrotactile actuators were co-located with the soft actuators corresponding to the three digits outfitted with BioTac tactile sensors for the thumb, index and little fingers (Vib_T_, Vib_I_, and Vib_L_ in Fig. 6). More information characterizing the vibrotactile stimulator is available in Ref.^64^.

### Session 1: experimental protocol for single object transportation

The first session of experiments lasted approximately 90 min and consisted of training the subjects to control individual EMG signals from one muscle group at a time (Fig. S2) and to integrate haptic feedback into their grasp control strategies for a single object at a time. To counterbalance against learning effects, half of the subjects transported the ball first while the other half transported the block first (Table S2). Full details of this session of experiments are included in the Supplemental Document (Table S3).

### Session 2: simultaneous control of forces applied to two grasped objects

#### Simultaneous EMG training—efferent only

The second session of experiments, which occurred on a subsequent day, began with training the human subjects to control their EMG signals simultaneously using Simulink. After adjusting the EMG gains for the comfort of each subject, they were asked to follow a trapezoidal pattern displayed on a computer monitor with both their EMG signals simultaneously (Fig. S2). This phase occurred prior to integration of haptic feedback.

#### Training to integrate multiple haptic feedback channels into simultaneous EMG control strategy

Next, haptic feedback from the soft robotic armband was integrated into the same simultaneous tracking task, where the pressure of each soft actuator increased proportionally to the corresponding EMG signals. The interim resort to EMG (as opposed to pressure information from the BioTac sensors) was necessary because the subjects had not progressed to using the physical system at this stage. Mapping haptic pressure to the corresponding EMG signal is a predictive form of biofeedback that has been previously shown to be effective to control the grip force of a Michelangelo prosthetic hand^2^. During this part of the training, subjects were able to see their normalized EMG signals on the computer monitor and the upper object-break threshold so they could make a correlation between the EMG signals and haptic sensations (Fig. S2). Corresponding vibrotactile stimulators were activated for one second if a person exceeded one or both upper break thresholds, indicating that one or both objects would have been broken by the robotic hand.

#### Training for simultaneous control with the robotic hand

Next, the subjects were trained with the robotic arm and hand to grasp and transport both objects at the same time without breaking or dropping them and to deliver both objects to the correct locations. From this stage on, the haptic feedback experienced by subjects was sourced from the measured BioTac fingertip force sensors (5–7), (Figs. 5, 6, 7).

The robotic hand was initially positioned above the ball and block (Fig. 2A). The subjects simultaneously flexed both muscle groups to appropriately increase the grasp forces on both objects at the same time without breaking either object (Fig. 4A–D). Once the subject was satisfied with the grip forces applied to the objects using haptic and visual feedback, they pressed the enter button on the keyboard in front of them to commence arm movement toward the delivery locations. The arm moved autonomously with an average speed of 0.1 m/s to provide a well-controlled experiment across all 12 test subjects. The arm motion was continuous and did not stop, requiring the subjects to use precise timing to release the objects into the correct bins. The subjects were asked to deliver the block into the first bin and then the ball into the second bin (Fig. 2A–D). However, it is worthwhile to mention that it is possible (but more difficult) to deliver both objects simultaneously with the developed simultaneous control algorithm (Fig. S4, Movie S7). Due to the constant speed of the robotic arm and different object delivery locations, subjects were trained to perform the delivery with one object at a time (Movie S3).

**Figure 8:**
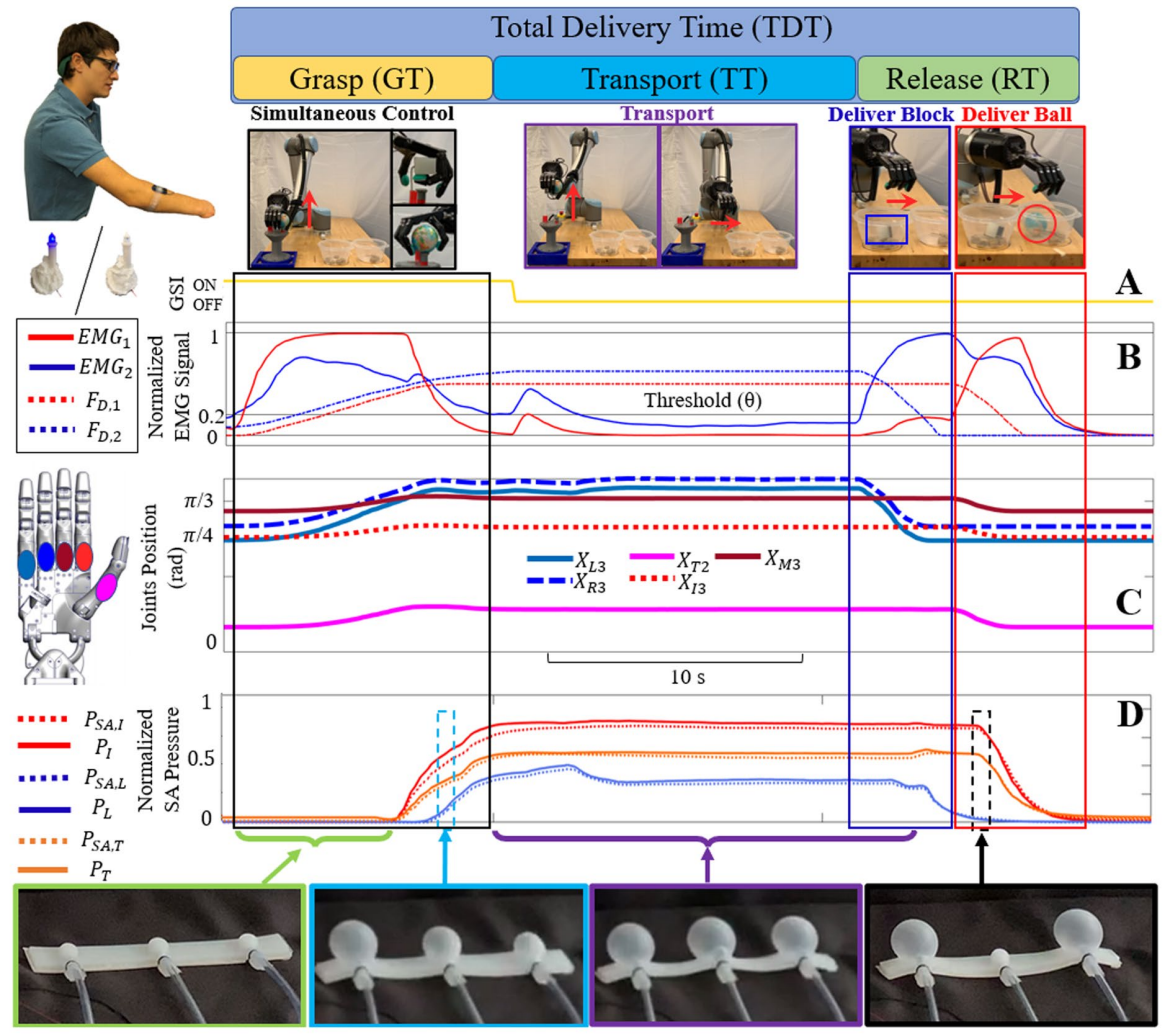
Exemplary trial showing afferent and efferent signals during grasp, transport and delivery of two objects simultaneously (see also Fig. 2A–D for photographic correlation to the sequence of actions). (**A**) The grasp state indicator (GSI) showing open/close state of the hand. (**B**) EMG signals were simultaneously co-activated at the beginning of the trial to grasp both objects. A rapid co-contraction of EMG signals toggled the GSI to switch from the opening mode to the closing mode of the hand. Then EMG_2_ was increased followed by EMG_1_ to deliver the block and ball to their respective locations. (**C**) Selected joint angles of the hand increased and then decreased as the two grasps of the hand were closed and opened to grasp, transport, and release the objects. (**D**) Haptic feedback signals increased after the objects were contacted to stably grasp both the ball and block. Then the pressure levels in the soft robotic armband decreased sequentially as the two objects were delivered.

However, all subjects were trained to grasp both objects simultaneously at the beginning of each trial (Fig. 4A–D) to investigate the capacity for simultaneous grasp control of a dexterous artificial hand. Illustrative data showed a person simultaneously flexing two muscle groups (EMG_1_ and EMG_2_, Fig. 8B) to close the two different grasps of the hand onto the two objects simultaneously. Joint angles of the fingers and thumb increased (Fig. 8C) until the objects were contacted. Then the fingertip forces increased, prompting corresponding haptic feedback pressures to increase and provide tactile sensations to the subject regarding the simultaneously controlled fingertip forces applied to both objects (Fig. 8D). A rapid co-contraction of EMG signals during transportation toggled the GSI and the open/close mode of the hand (Fig. 8A). The subject next flexed EMG_2_ to deliver the block and then flexed EMG_1_ to deliver the ball (Fig. 8B).

#### Impact of haptic and visual feedback on simultaneous grasp force control

After all training, the experimental protocol with the robotic system was designed to systematically evaluate the impact that haptic (H) and visual (V) feedback had upon this complex simultaneous control task. The full factorial design included four possible feedback conditions combining these two independent variables: haptic and visual feedback (HV), only haptic feedback (H), only visual feedback (V), and neither haptic nor visual feedback (**∅**). Occluding visual feedback was accomplished by erecting an opaque curtain to block view of the grasp portion of the experiments; the subjects were always able to see the delivery locations as well as the GSI.

Testing the impact of haptic and visual feedback on simultaneous control of a dexterous artificial hand was done first with a non-randomized progression of the trials (Table S5) with half of the subjects deprived of visual feedback first while the other half of the subjects were allowed to view the robotic system first to counterbalance against learning effects (Table S2). This procedure was followed by a repeat session with a pseudo-random organization of the vision and haptic feedback independent variables (Table S6). The culmination of Session 2 experiments had subjects perform 16 trials of the simultaneous grasp control and transportation task, where each of the four possible combinations of independent variables (haptic/visual feedback), arranged in a pseudo-random order, were implemented four times by all of the 12 subjects. Randomization of the order of trials was done in MATLAB using the randperm function. A three factor ANOVA was performed using MATLAB. The three independent variables were the human subjects, visual, and haptic feedback. A trial was considered a success if neither failure (drop, break) occurred at any time during the trial. After completing all experiments subjects were also asked a single question to subjectively rate how helpful the haptic and visual feedback was to perform the tasks on a scale of 0–5. A non-parametric Mann–Whitney U-test was performed to assess the significance of these subjective ratings.

#### Impact of haptic and visual feedback on total delivery time and simultaneity

To quantify the impact that haptic and/or visual feedback had upon the time to complete the task for each subject during the experiments, the TDT metric was defined as:

8 $$TDT = GT + TT + RT,$$

where GT was the time needed to grasp the object(s), TT was the time to transport the object(s), and RT was the time required to release the object(s) (Figs. 3, 8).

When grasping both objects simultaneously, GT was defined as the amount of time from when the first EMG signal rose above the minimum threshold (θ) until both desired forces stabilized at their maximal values prior to initiation of the transportation sequence. TT was the time required by the robotic arm to transport the objects to the delivery locations. RT was defined as the amount of time from when the first EMG signal rose above the activation threshold until both desired forces returned to zero (Fig. 3A).

During the initial grasp portion of the experiments (GT, Figs. 2A, 4A–D), subjects were trained to go at their own pace and to prioritize successful completion of the task without breaking or dropping either object. Three factor ANOVA was performed to investigate any statistically significant differences that the subjects, haptic, and visual feedback had upon the TDT (8) and simultaneity metric (1) during the simultaneous control experiments.

## Supplementary Information

Supplementary Legends.

Supplementary Information 2.

Supplementary Video 1.

Supplementary Video 2.

Supplementary Video 3.

Supplementary Video 4.

Supplementary Video 5.

Supplementary Video 6.

Supplementary Video 7.

## Data Availability

The data presented in this paper will be made available upon reasonable request.
