# Supplementary material for: Multichannel haptic feedback unlocks prosthetic hand dexterity
Source: Sci Rep. 2022 Feb 11;12:2323. doi: 10.1038/s41598-022-04953-1 (PMC8837642; doi:10.1038/s41598-022-04953-1)
Supplement: Supplementary file 1 — Supplementary Legends. [file 41598_2022_4953_MOESM1_ESM.pdf]

SUPPLEMENTARY MATERIALS

Fig. S1. Trajectory planning for simultaneous grip force control.
Fig. S2. Training protocol for efferent control.
Fig. S3. Single Object Delivery Success and Failure Rates.
Fig. S4. Overview of the dexterous object transportation task with two objects with emphasis on robotic hand actions for the situation with a simultaneous grasp and simultaneous delivery.
Fig. S5. Comparison of total delivery time required to grasp, transport, and release single object with haptic feedback and with no haptic feedback.
Fig. S6. Success and failure rates of the simultaneous object delivery task during the non-randomized experiments.
Fig. S7. Illustrative data showing objects being broken in the absence of haptic feedback.
Fig. S8. Comparison of total delivery time required to grasp, transport, and deliver two objects simultaneously with the four possible combinations of independent variables.
Fig. S9. Comparison of the simultaneity metric under the four-time windows, t_1, t_2, t_3, and t_4.
Fig. S10. The robotic arm performing two functionalities simultaneously: grasping and transporting a ball with a tripod grasp while flipping a light switch with the little finger.
Fig. S11. Simultaneous EMG control of two different grip functions of the Shadow Hand.

Table S1. Session 1: Randomized haptic (H) and no haptic (NH) trial sequence for the single-object transportation experiments.
Table S2. Grouping of subjects.
Table S3. Sequence of Experiments During Session 1 (transporting one object at a time).
Table S4. Session 1: Guessing game using haptic feedback only.
Table S5. Session 2: Organization of non-randomized experiments.
Table S6. Session 2: Organization of randomized experiments.

Movie S1. Single object transportation of the ball.
Movie S2. Single object transportation of the block.
Movie S3. Simultaneous object transportation of the ball and block.
Movie S4. Function of the soft robotic armband for multichannel haptic feedback.
Movie S5. Simultaneously grasping a card and unscrewing a lid.
Movie S6. Simultaneously grasping a ball while toggling a light switch.
Movie S7. Simultaneously grasping and delivering both the ball and block.
