# Supplementary material for: Multichannel haptic feedback unlocks prosthetic hand dexterity
Source: Sci Rep. 2022 Feb 11;12:2323. doi: 10.1038/s41598-022-04953-1 (PMC8837642; doi:10.1038/s41598-022-04953-1)
Supplement: Supplementary file 2 — Supplementary Information 2. [file 41598_2022_4953_MOESM2_ESM.pdf]

Supplemental Document
Multichannel haptic feedback unlocks prosthetic hand dexterity

1. Trajectory Plans to Simultaneously Grasp Two Different Objects

Two objects were selected for the Shadow Hand to grasp individually or simultaneously: a ball and a block, as these are commonly shaped items. The elastic ball was manipulated with Grasp 1 using the thumb (T), index (I), and middle (M) fingers (tripod grasp). The block was made of Ecoflex-30 (Smooth-On, Inc., Macungie, PA, USA), and was manipulated with Grasp 2 using the little (L) and ring (R) fingers opposed to the palm. For consistency, both objects were placed on a stand that was designed using SolidWorks (Trimech, VA) and printed with an Ultimaker3 (Ultimaker B.V. Utrecht, Netherlands) using polylactic acid. The Ecoflex block had a small metal plate inserted into the base so that it could be consistently placed in the exact same location on the test stand, ensuring repeatable test conditions across all subjects (Fig. 2A).

To create suitable grasp trajectory plans for each object, the Shadow Hand was placed over the ball and block so that the thumb, index, and middle fingers could grasp the ball while the little and ring fingers could grasp the block (Fig. 2A). BioTac values for the thumb, index finger and little finger were monitored to ensure a firm grasp on the objects while the starting and final joint angles were recorded in Robot Operating System (ROS) with the rosbag command.

The recorded initial and final joint angles were used to create the joint space trajectory polynomials. These joint angles were imported into MATLAB and the polynomials were created for each joint of the Shadow Hand (Fig. S1A). The initial and final joint positions were used to solve a set of 5th order polynomials for each grasp with a vector normalized to the same 0-1 scale as the users' normalized EMG signals[49,59]. This enabled the two EMG signals to synchronously control two different grasps, each of which were comprised of multiple DOFs. A 5th order polynomial was used for each joint because it yielded smooth motion profiles. Joint angles for the ring and little fingers smoothly changed from initial to final postures (Fig. S1B) as did the thumb, index, and little fingers (Fig. S1C).

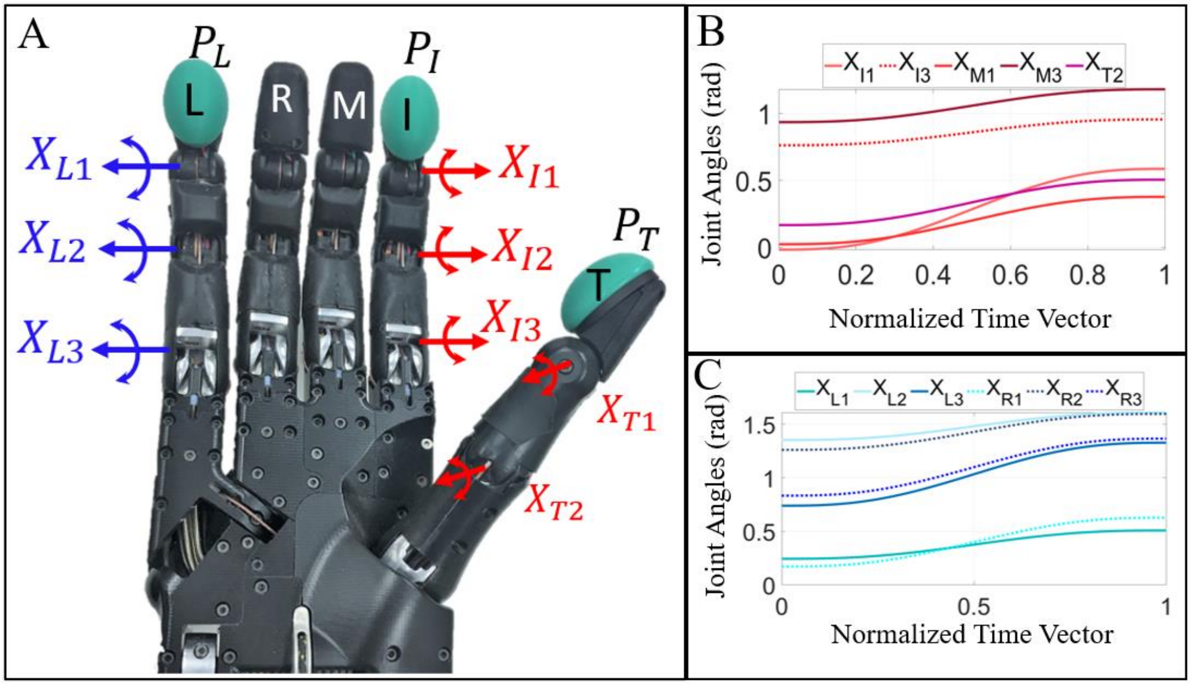

Fig. S1. Trajectory planning for simultaneous grip force control. (A) Axes of revolution used the digits of the Shadow Hand. P_T, P_I, and P_L are the pressures measured by the BioTac sensors on the thumb (T), index (I), and little (L) fingers, respectively. The ring (R) finger was used with Grasp 2 for the block and the middle (M) finger was used in the tripod Grasp 1 for the ball. (B) Selected trajectory plans for the thumb, index and middle fingers to grasp the ball, Grasp 1. (C) Selected trajectory plans of the joint angles for little and ring fingers to grasp the block, Grasp 2.

The inputs for the trajectory plan for each grasp G ∈ 1, 2 are based on the corresponding grasp force error ($e_{F,G}$) between the measured and desired forces (Fig. 6):

$$e_{F,G} = F_{D,G} - F_G \,. \tag{S1}$$

F_G , F_{DG} are the measured and the desired forces, respectively, for grasp G. The measured force on the robotic hand is calculated as the proportionally scaled pressure measurement (P_K) from the corresponding BioTac SP on the Shadow Hand, where K ∈ I, L, T corresponds to the index finger (I), little finger (L) and thumb (T).

These two force control errors were proportionally amplified by gains ($\beta_{P,G}$) to form the desired angular position input vectors ($\overrightarrow{X_{D,G}}$) to two position control loops for all the joints within the two grasps. Angular position error vectors ($\overrightarrow{E_{X,G}}$) for each joint in the two grasps were defined as:

$$\overrightarrow{E_{X,G}} = \overrightarrow{X_{D,G}} - \overrightarrow{X_G} = \overrightarrow{\beta_{P,G} Q_G E_{F,G}} - \overrightarrow{X_G} \,. \tag{S2}$$

$\overrightarrow{X_G}$ are the measured angular positions of the joints in grasp G, and

$$\overrightarrow{E_{F,G}} = \begin{bmatrix} 1 & e_{F,G} & e_{F,G}^2 & e_{F,G}^3 & e_{F,G}^4 & e_{F,G}^5 \end{bmatrix}^T \,. \tag{S3}$$

Matrices $Q_1 \in R^{13x6}$ and $Q_2 \in R^{9x6}$ contain the coefficients of the quintic polynomials for each grasp plan:

$$Q_1 = \begin{bmatrix} a_{0,I1} & \cdots & a_{5,I1} \\ \vdots & \cdots & \vdots \\ a_{0,I3} & \cdots & a_{5,I3} \\ a_{0,M1} & \cdots & a_{5,M1} \\ \vdots & \cdots & \vdots \\ a_{0,M3} & \cdots & a_{5,M3} \\ a_{0,T2} & \cdots & a_{5,T2} \end{bmatrix}, \quad Q_2 = \begin{bmatrix} a_{0,L1} & \cdots & a_{5,L1} \\ \vdots & \cdots & \vdots \\ a_{0,L3} & \cdots & a_{5,L3} \\ a_{0,R1} & \cdots & a_{5,R1} \\ \vdots & \cdots & \vdots \\ a_{0,R3} & \cdots & a_{5,R3} \end{bmatrix} \tag{S4}$$

The ‘a’ coefficient in the Q_G matrices were planned to enable subjects to grasp the two different objects while simultaneously guaranteeing smooth joint velocity and acceleration profiles (Fig. S1).

2. SUPPLEMENTAL METHODS FOR SESSION 1: MANIPULATING ONE OBJECT AT A TIME

The first session of experiments occurred during the first day that the subjects participated in experiments in the lab. This session of experiments incrementally trained subjects to integrate haptic feedback into their artificial hand control strategies prior to the more complex simultaneous control experiments during session 2, which occurred on a subsequent day. Experiments with the robotic system during session 1 of experiments were focused on training the subjects to grasp and transport a single object at a time, without breaking or dropping it, using EMG control with and without haptic feedback from the soft robotic armband (Table S1). Vision was not blocked during these object transportation experiments in Session 1. However, vision was blocked at the culmination of session 1 to test the extent to which subjects could perceive what object(s), if any, were in the grasp of the hand when vision was occluded during 16 additional grasp tasks that occurred after the object transportation experiments (Table S4).

Prior to these robotic experiments, initial EMG training during session 1 was broken into three parts: switching grasp operational mode (open/close), EMG training without haptic feedback (Fig. S2A-C), and EMG training with haptic feedback.

2.1 EMG Training to Switch Hand Operational Mode

In the first part of the training, EMG surface electrodes were placed on the flexor carpi radialis and extensor digitorum communis muscles of each subject's forearm and the gains were adjusted so that the subjects could comfortably flex their muscles and reach maximum permissible values, normalized on a scale from 0 to 1. After the gains were adjusted, the subjects were asked to rapidly co-contract several times to explain the mechanism for switching between opening and closing the hand. It was also foreshadowed that co-contracting less rapidly would allow another option: to simultaneously and proportionally control the forces applied to two objects grasped at the same time (during session 2 of experiments).

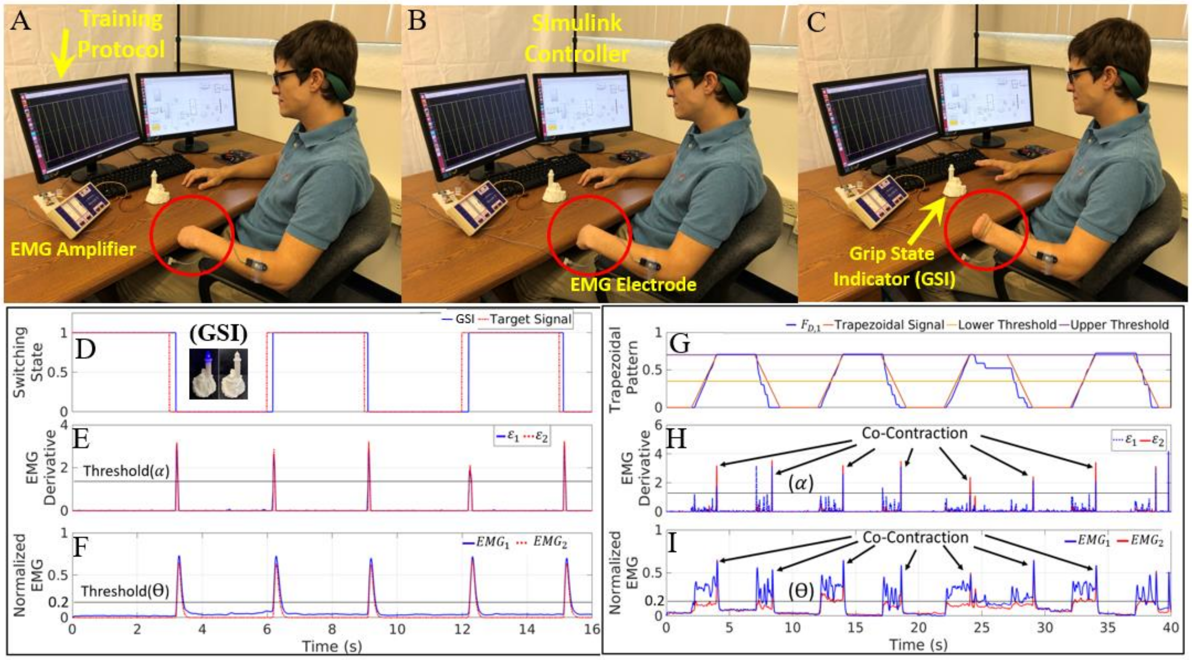

Fig. S2. Training protocol for efferent control. (A) Subject 12 going through the training protocol prior to integration of haptic feedback. (B) He practiced flexion and (C) extension to track the trapezoidal trajectory displayed on the monitor with his EMG signals. (D) EMG training to switch the hand operational state between opening and closing via a rapid co-contraction to track the target signal. (E) The positive portions of the EMG derivatives used by the algorithm to distinguish between simultaneous control of grasp forces applied to two objects and the mode switching of the hand. (F) Corresponding EMG signals to switch modes. (G) In the next phase of training, subjects were trained to make their desired force track the trapezoidal target signal. This was done first without and then with the soft robotic armband to learn to integrate bimodal multichannel haptic feedback. They observed the upper and lower thresholds displayed on the monitor as an indicator of the minimum and maximum forces to apply on the objects. A desired force below the lower threshold would probably drop an object while a force above the upper threshold would break the object. (H) Corresponding positive portions of the EMG derivatives and the (I) EMG signals required to track the trapezoidal trajectories.

The subjects were comfortably seated in front of a computer monitor to view two signals shown on the screen: a 1/6 Hz square wave, and the open/close grasp mode of the hand. Subjects were asked to make their switching signal

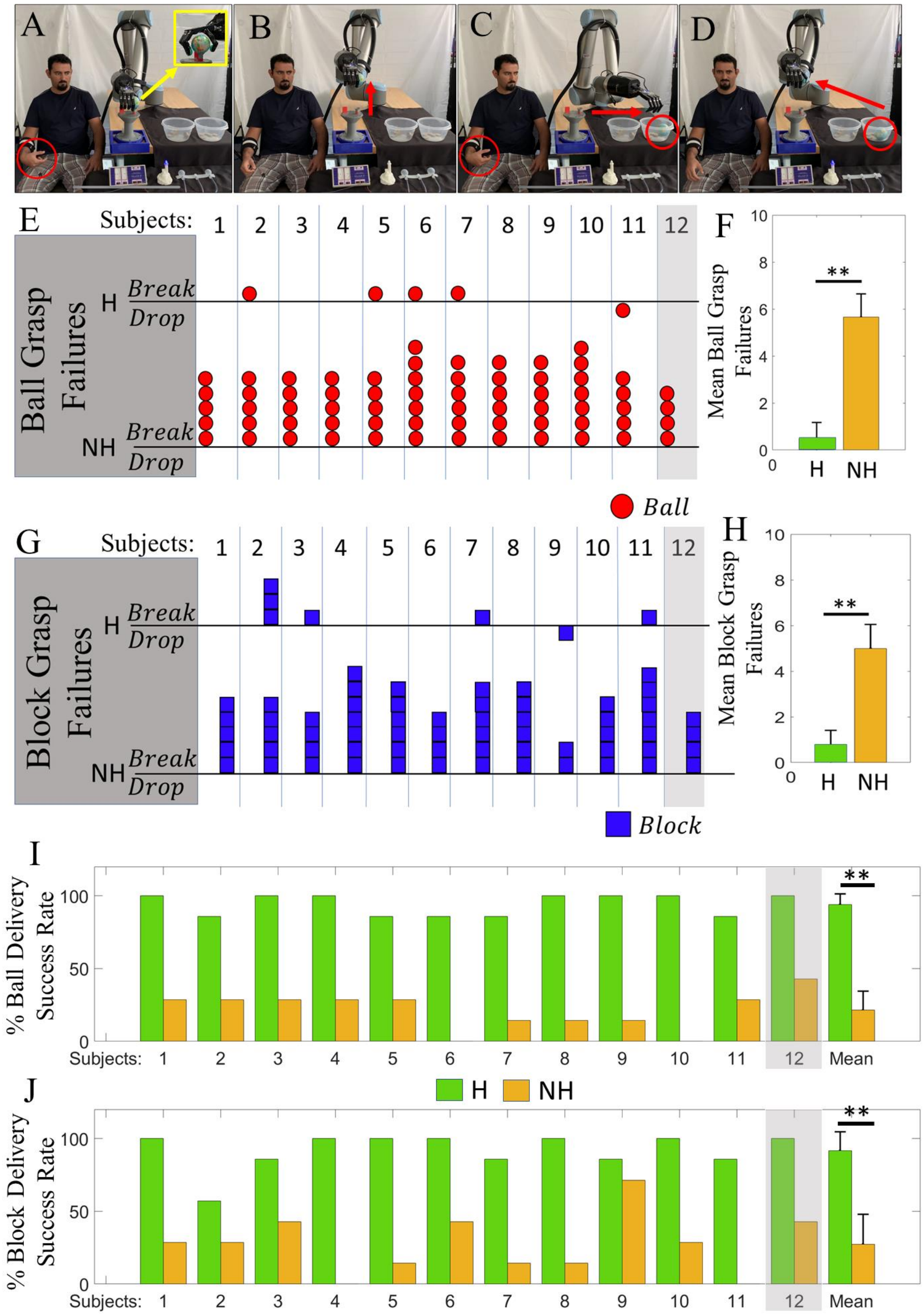

Fig. S3. Single Object Delivery Success and Failure Rates. (A) The subject flexed EMG_1 to grasp the ball, and (B) then co-contracted rapidly to switch the grasp mode. (C) The subject flexed EMG_1 again to deliver the ball into the bin and (D) the arm returned to repeat the cycle. (E) The total number and type of failures for each subject and (F) their overall averages during the single object transportation tasks with Haptic 'H' feedback and No Haptic 'NH' feedback. (G) Failures with the block by subject and (H) their composite averages. (I) The success rates for the 12 test subjects to transfer the ball and (J) block, one at a time, with and without haptic feedback. Limb absent subject 12 is highlighted grey. ** $p<0.01$.

follow the square wave signal for one minute as it repeatedly changed between 0 and 1 by rapidly co-contracting their muscles on the rising and falling edges of the square wave (Fig. S2D-F); see also Fig. 5B and eqns (2)-(4).

2.2 EMG Training Without Haptic Feedback

Subjects were next trained to proportionally control their individual EMG signals, one at a time, by following a trapezoidal pattern created in Simulink that was displayed on a computer monitor (Fig. S2 D-F). They were asked to follow the trapezoidal pattern with EMG from E_1 first, and then with EMG from E_2. The grasp state indicator (GSI) was placed in front of the subjects at all times to indicate the mode of the hand (opening or closing).

2.3 Training to Integrate Haptic Feedback into EMG Control

Next, the subjects were asked to repeat the same experiment described in section 2.2 (Fig. S2 D-F) with haptic feedback from the soft robotic armband so that they would become familiar with the artificial sensations of touch. The soft robotic armband was placed onto subjects' upper arms using a stretchable arm brace (KYUSports) to hold the robotic armband in place. In this part of the training, subjects were asked to repeat the same EMG training with the trapezoidal patterns; the only added feature was that their EMG signals were used to proportionally control the pressures in the soft actuators integrated into the armband. This predictive form of biofeedback[2] was necessary since the subjects had not yet progressed to controlling the robotic hand at this point. The trapezoidal tracking training task was similar to the control they would need to implement during the actual experiment with the robotic arm and hand: closing the hand to grasp the object and increase the grip force to prepare for lifting and transporting the object, then decreasing the grip force and releasing the object into the correct delivery location.

During these experiments, the same trapezoidal signal and their EMG signals were displayed on the monitor in addition to two constant horizontal lines. The lower line was set at 0.35, as this was an estimate of the minimum grip force required to lift the objects without dropping them. The upper line was a breaking threshold that was set at 0.7 to provide a moderate challenge to grasp and transport the objects without breaking them.

The subjects were asked to follow the trapezoidal pattern once again but this time they were trained to stop when the amplitude of their signal (and the corresponding haptic feedback) was slightly below the breaking threshold (0.7), as this was the safe range to perform the object transportation experiments with the robotic system. After following the signal ten times with one EMG signal they repeated this with the other. If either EMG signal exceeded the upper threshold of 0.7, the corresponding vibrotactile stimulators on the armband were actuated for 1 second to simulate a broken object.

2.4 Training with the Robotic System

Next, subjects were trained on the robotic system. During the training phase, the computer monitor showing the EMG signals and thresholds was still placed in front of the subjects as a reference during the final stage of training. After the subject became confident to grasp and release the objects without breaking them, they were given two practice runs with the robotic arm to grasp and transport the ball and block, one at a time, to the corresponding delivery locations (Fig. S3 A-D).

2.5 Single Object Transportation Experiments with the Robotic System

After the training runs with the robotic system, subjects next performed a total of 14 trials with each object (Fig. S3 A-D). Half the trials had haptic feedback disabled, which were sequenced in a pseudo-random manner; each subject followed the same randomized pattern (Table S1). Randomization of the haptic feedback variable was done in MATLAB using the randperm function; vision of the single object transportation tasks was never blocked as in the simultaneous experiments in session 2. After the subject was comfortable with the initial grasp force applied to the object, they were asked to press the 'enter' key on the keyboard in front of them to trigger arm movement, which occurred continuously; the subject had to use precision timing to drop the object into the correct bin because the arm did not pause during the motion (Movies S1, S2). To produce a well-controlled experiment, the robotic arm moved autonomously during these experiments with an average end effector speed of 0.1 m/s.

2.6 Grouping of Subjects to Counterbalance Learning Effects

For experiments with the robotic system in session 1, half of the subjects performed the transportation of the ball first while the other half of the subjects transported the block first in order to counterbalance learning effects (Table S2). Depending upon the group, half of the subjects performed the sequence in Table S1 with the ball first while the other half of the subjects did this with the block first.

2.7 Using Haptic Feedback to Determine Which Object(s) Were Grasped: Guessing Game

After finishing all the trials with transporting the ball and the block, one at a time, an opaque curtain was drawn to occlude the subject's view of the robotic hand. Once the view of the hand was occluded, they were asked to guess which object, if any, was grasped by the hand using haptic feedback alone. In this task there were 16 trials divided evenly amongst the 4 possible options of {1} ball, {2} block, {3} both objects, {4} neither object. The sequence of these trials for determining the combination of objects, if any, during the guessing game was again randomized using the randperm function and all the subjects followed the same sequence (Table S4). All experiments performed in session 1 are listed in Table S3.

Table S1. Session 1: Randomized haptic (H) and no haptic (NH) trial sequence for the single-object transportation experiments.

**Trial #**	**Haptic (H)**	**No Haptic (NH)**
**1**	✔	
**2**		✔
**3**	✔	
**4**	✔	
**5**		✔
**6**	✔	
**7**	✔	
**8**		✔
**9**		✔
**10**		✔
**11**	✔	
**12**		✔
**13**	✔	
**14**		✔

Table S2. Grouping of subjects. The 12 subjects were organized into four groups to counterbalance against learning effects. All subjects performed the same experiments, just the sequencing of experiments was organized to counterbalance against learning effects. Listed below is the organizational sequence of the independent variables for each group.

	Session 1: One Object at A Time		Session 2: Simultaneous; Non-randomized	
Group 1	Object 1 (Ball)	Object 2 (Block)	Vision	No Vision
Group 2	Object 1 (Ball)	Object 2 (Block)	No Vision	Vision
Group 3	Object 2 (Block)	Object 1 (Ball)	Vision	No Vision
Group 4	Object 2 (Block)	Object 1 (Ball)	No Vision	Vision

Table S3. Sequence of Experiments During Session 1 (transporting one object at a time).

Session 1: Grasp, Transport, and Deliver One Object at a Time
Training to Switch Between Grasp Modes
EMG Training with Computer Monitor Display using EMG_1 and EMG_2 (No Haptic Feedback; Fig S2A-C)
EMG Training with Computer Monitor Display using EMG_1 and EMG_2 (With Haptic Feedback)
Robotic System Training (Fig. S3A-D)
Robotic System Testing: 14 Trials implemented with each object: (7 with Haptic and 7 without Haptic)/object
Guessing Game: Use haptic feedback to determine which object(s) were grasped. (Without Vision, see Table S4)

3. Supplemental Methods for Session 2 Experiments: Simultaneously Grasping and Transporting Two Objects Without Breaking or Dropping Them

On a subsequent day, each subject performed 16 non-randomized (Table S5) and then 16 pseudo-randomized experiments (Table S6) to simultaneously control the grip forces applied to both objects (Fig. 2A-D), (Fig. S4; Movie S3). Note that Fig. S4 shows the situation where both objects were delivered to their respective delivery locations simultaneously, which is another possible method to successfully complete the task (Movie S7).

Prior to experiments with the physical system, simultaneous EMG control training occurred as described in the main document. Then, simultaneous control experiments were performed with the robotic system first in a non-randomized fashion and then in a pseudo-randomized manner with four repetitions of each of the four possible combinations of the two independent variables (Haptic and Visual feedback). At this time, grouping of subjects were

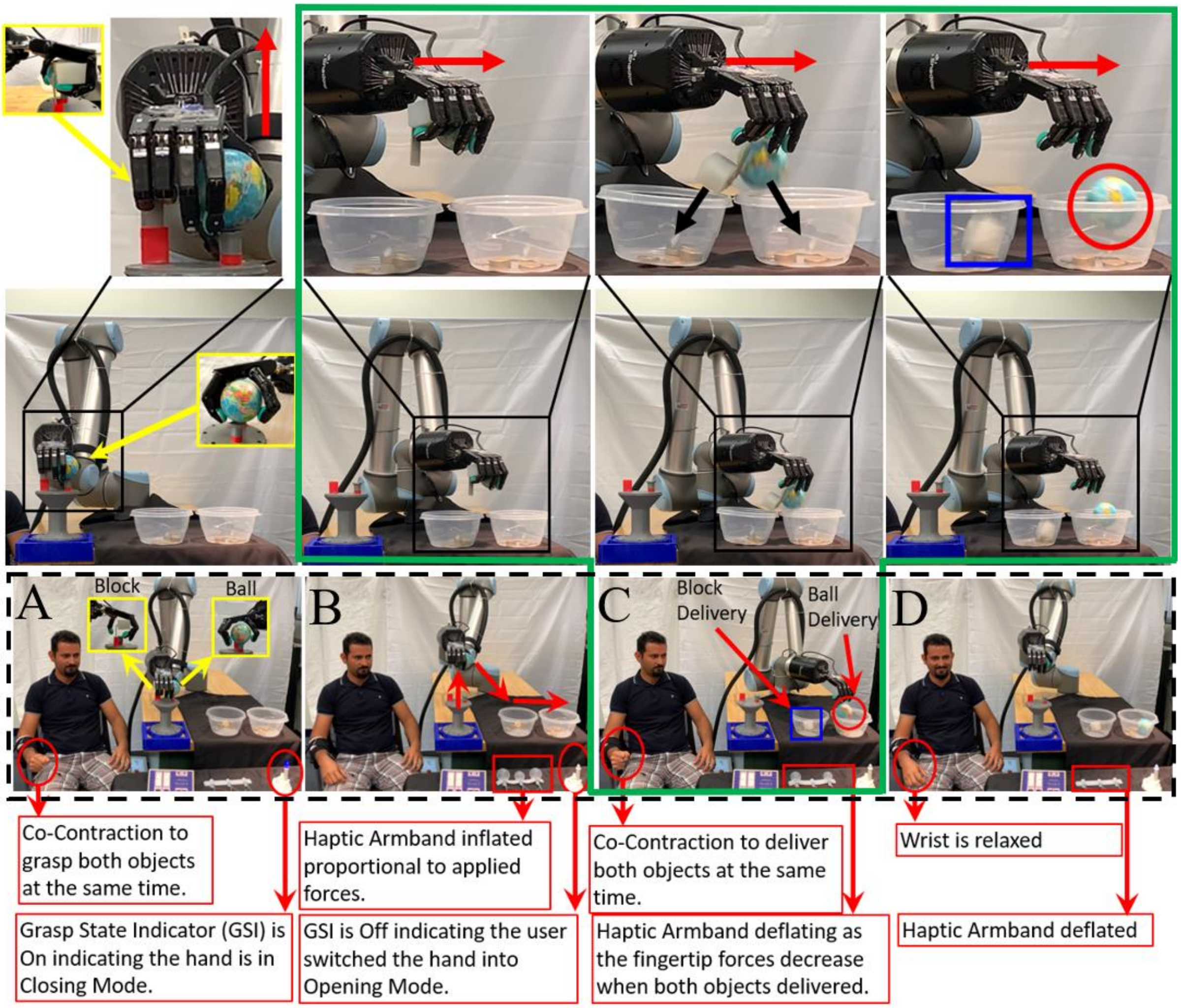

Fig. S4. Overview of the simultaneous dual-object transportation task with emphasis on robotic hand actions for the situation with a simultaneous grasp and simultaneous delivery. (A) Human Subjects were asked to make sure that the hand was in the closing mode and they needed to skillfully co-contract to grasp both objects at the same time without breaking them. (B) Shadow Hand lifted both objects and the subjects maintained appropriate grip forces to avoid dropping either object. For illustrative purposes, note that the haptic armband had the actuators inflated proportionally to the grip forces. (C) The subject contracted both muscle groups simultaneously to deliver the block and ball into their respective bins. (D) The subject relaxed as the arm returned to the initial location to repeat the experiment.

subdivided again were to produce four different groups of subjects in total. Subjects in Group 3 (Table S2) performed the non-randomized trials with vision available during the first half of the 16 trials (Table S5): trials 1-4 were conducted with both haptic feedback and vision available (HV), while trials 5-8 were performed without haptic feedback, but with vision of the robotic system (V). Trials 9-12 were conducted with haptic feedback but vision of the robotic arm during the grasp phase was occluded by the curtain (H). Trials 13-16 were conducted with neither haptic nor visual feedback (ϕ). Subjects in Group 4 (Table S2) performed the same trials, but without vision of the system first, which is to say they performed trials 9-16 first, then trials 1-8 listed in Table S5. All subjects performed the same experiments, just the sequencing of when the experiments were performed was organized to counterbalance against learning effects with respect to vision of the system during the non-randomized experiments.

After the non-randomized experiments, subjects performed the same tasks organized in a pseudo-randomized fashion (Table S6), where each combination of independent variable (Haptic/Visual) was tested four times by each subject.

4. Supplemental Results For Session 1: Single Object Control

4.1 Session 1: Single Object Transportation Success and Failure Rates

Subjects were able to grasp (Fig. S3 A), lift (Fig. S3 B), transport (Fig. S3 C), and deliver the ball (Fig. S3 D), and the block, one at a time (Movies S1, S2). The success rate of each subject was significantly higher with haptic feedback than without haptic feedback while delivering both the ball and block, one object at a time (Fig. S3 (I-J)). In this case, a trial was considered a success if no failures, such as breaking or dropping the object, occurred at any time during the task. The average success rate with haptic feedback was 94.3% ± 7.4% with the ball and 91.6% ± 12.9% with the block, while the average success rate without haptic feedback worsened substantially to 21.4% ± 12.8% with the ball and 27.4% ± 20.6% with the block. Subjects 1, 4, 8, 10, and 12 (who has a congenital absence of his hand) were able to perform all trials without any failures when they had haptic feedback available.

All subjects had significantly less incidents of failure when they had haptic feedback from the robotic armband in comparison to the trials without haptic feedback (Fig. S3 E-H). All 12 subjects broke both objects multiple times during trials without haptic feedback, which was the most common mode of failure during these trials.

Table S4. Session 1: Guessing game using haptic feedback only. Pseudo-random sequence of four possible combinations of objects grasped to ascertain how well subjects could integrate haptic feedback into their sensorium while vision was occluded.

Trial #	Ball	Block
1	✓	
2		
3	✓	✓
4	✓	
5		
6	✓	✓
7	✓	
8		✓
9		
10	✓	✓
11		✓
12	✓	
13		✓
14		
15	✓	✓
16		✓

Table S5. Session 2: Sequence of non-randomized experiments performed by Group 3. The four possible combinations of two independent variables (Haptic Feedback and Vision) were tested by the twelve subjects to gain experience prior to randomized trials.

Trial #	Haptic (H)	Vision (V)	Symbol
1	✓	✓	HV
2	✓	✓	HV
3	✓	✓	HV
4	✓	✓	HV
5		✓	V
6		✓	V
7		✓	V
8		✓	V
9	✓		H
10	✓		H
11	✓		H
12	✓		H
13			ϕ
14			ϕ
15			ϕ
16			ϕ

The ANOVA showed that the haptic feedback made a statistically significant impact for the success and failure data during the single object transportation experiments with each object ($p<0.01$).

4.2 Session 1: Guessing Game: Haptic Feedback Enabled Subjects to Determine Which Object(s) Were Grasped When Vision was Occluded

When view of the robotic hand was blocked, subjects were able to use haptic feedback alone to determine which object(s), if any, were grasped by the hand. Subjects were able to interpret haptic feedback from the soft robotic armband with high success rates. There were twelve subjects, each of whom made 16 guesses producing 192 total guesses. Subjects correctly guessed that the ball was grasped 95.8% ± 9.7%. For the other three cases (block, both objects, and neither object) subjects had the same overall rate of correct guessing of 97.9% ± 7.2%. In other words, there were two total instances of incorrectly identifying that the ball was grasped. All three other cases had one instance of incorrect identification.

Table S6. Session 2: Organization of randomized experiments. The four possible combinations of two independent variables (Haptic Feedback and Vision) were tested by the twelve subjects.

Trial #	Haptic (H)	Vision (V)	Symbol
**1**	✔	✔	HV
**2**		✔	V
**3**	✔		H
**4**	✔		H
**5**			ϕ
**6**		✔	V
**7**	✔	✔	HV
**8**			ϕ
**9**		✔	V
**10**	✔		H
**11**	✔		H
**12**			ϕ
**13**	✔	✔	HV
**14**			ϕ
**15**	✔	✔	HV
**16**		✔	V

4.3 Session 1: Total Delivery Time for Both Objects, One at a Time

Fig. S5 shows a comparison of total time required to grasp, transport, and deliver single object with and without haptic feedback. Here, the grasp time (GT), transportation time (TT), and release time (RT) are added together to produce the total delivery time (TDT) metric (8) for each object that was delivered

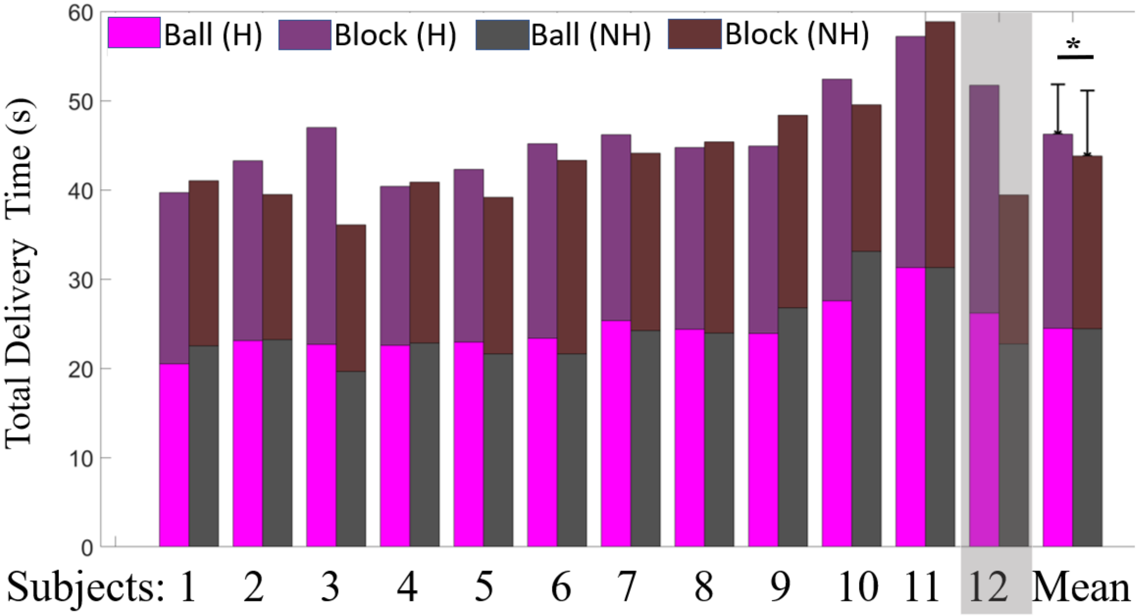

Fig. S5. Comparison of total delivery time required to grasp, transport, and deliver objects one-at-a-time with haptic feedback (H) and with no haptic (NH) feedback. The gray highlight indicates the limb-absent subject 12. Haptic feedback significantly lengthened the total delivery time. * $p<0.05$.

one at a time (Fig. 3C). The TDT for the ball and block were then added together to indicate the entire time to deliver both objects, one at a time. The mean TDTs for the ball and the block when added together are 46.3 s ± 5.6 s for trials with haptic, and 43.8 s ± 7.3 s for the trials with no haptic feedback (Fig. S5). The ANOVA indicated that the haptic feedback significantly lengthened the TDT ($p<0.05$).

5. Supplemental Results for Session 2: Simultaneous Control

5.1 Simultaneously Controlling Forces Applied to Two Objects Grasped at the Same Time (Non-Randomized Experiments)

When simultaneously delivering both objects at the same time, haptic feedback was again the most important factor for all subjects to perform the task successfully (Fig. S6). The trials were considered a success if no failure (break or drop) occurred during the trial. The average success rate for the cases in the non-randomized test with haptic feedback were (91.6% ± 16.3% ball; HV), (89.6% ± 12.9% block; HV), (87.5% ± 16.8% ball; H), and (83.3% ± 19.5% block; H). Without haptic feedback, these success rates plunged significantly to (16.7% ± 22.2% ball; V), (25.0% ± 28.2% block; V), (14.6% ± 24.9% ball; Ø), and (14.6% ± 22.5% block; Ø), (Fig. S6 C-F).

The total number of failures that occurred with each subject was significantly less when haptic feedback was included in the operational mode of the system ($p<0.01$), (Fig. S6 (A, B)). The impact of visual feedback of the grasped objects was not significant ($p > 0.05$).

A typical example of breaking the objects for a trial without haptic feedback (Fig. S7) showed that the subject increased both EMG_1 and EMG_2 simultaneously to increase the grip forces on both objects at the same time (Fig. S7 (B)) prompting joint angles of the hand to close onto the objects (Fig. S7 (D)). Then a rapid co-contraction switched the operational state of the hand (Fig. S7 (A)) to open as it approached the delivery zones. Finally, the subject contracted EMG_2 to deliver the block and then contracted EMG_1 to drop the ball into the correct locations. Because the haptic feedback was disabled, the soft robotic armband pressures were all zero (Fig. S7 (E)) and the subject was unaware that the desired fingertip forces ($F_{D,1}$, $F_{D,2}$ in Fig. S7 C) and measured fingertip forces passed the upper 'break' thresholds (0.7) which resulted in a failed trial and a breakage of both objects.

5.2 Session 2: Impact of Haptic and Visual Feedback on Total Delivery Time (randomized experiments)

During the randomized experiments, the grasp time (GT), transportation time (TT) and the release time (RT) were added together to produce the TDT metrics (Fig. 3A). The mean delivery times required to deliver both objects are (32.7 s ± 4.2 s; HV), (35.4 s ± 6.3 s; H), (29.1 s ± 3.5 s; V), and (28.5 s ± 4.5 s; Ø). The mean TDT required to deliver the objects with haptic feedback was higher than the time required to deliver the objects without haptic feedback (Fig. S8); see also Fig. 3. Simultaneous object transportation was significantly faster than one object at a time, regardless of the feedback condition ($p<0.01$). We assessed significance between the TDTs of the single object and simultaneous object transportation task using an unbalanced ANOVA with the anovan function in MATLAB.

5.3 Simultaneity Metric is Impacted by Haptic and Visual Feedback During Randomized Experiments

It was clear that most subjects had the largest amount of simultaneity (1) when haptic and/or visual feedback were deprived (Fig. S9). The presence of haptic feedback served as an inhibitory cue to carefully increase the grip forces to acceptable levels. Subject 9 was the only person to show simultaneity during t_4 in the HV case while subject 11 was the only subject to exhibit simultaneity during t_4 with the H case. Congenital limb absent subject 12 (and subject 2) exhibited simultaneity during t_4 in both the V and ϕ cases but not in the HV or H cases, illustrating that they had effectively integrated haptic feedback into their simultaneous control strategies to avoid break failures. Only subjects 9 and 11 had a small level of simultaneity during t_4 when haptic feedback was enabled; all other subjects avoided this undesirable trait by properly interpreting the multichannel haptic feedback.

6. Additional Demonstrations of Simultaneous Control

To demonstrate the versatility of the simultaneous control approach, we showed that the same technique can be used in a variety of situations, such as to toggle a light switch with the little finger while grasping a ball with the index, middle fingers and the thumb (Fig. S10, Movie S6). In yet another application, two EMG signals can be used to simultaneously control any two functions, such as pinching a card between the index and middle fingers while using

the thumb and index finger to unscrew the lid of a water bottle (Fig. S11, Movie S5). In this demonstration, the signal processing for the EMG is the same, just the mapping of the EMG to the joint space is different, using the approaches outlined in[49,68] to unscrew the water bottle lid.

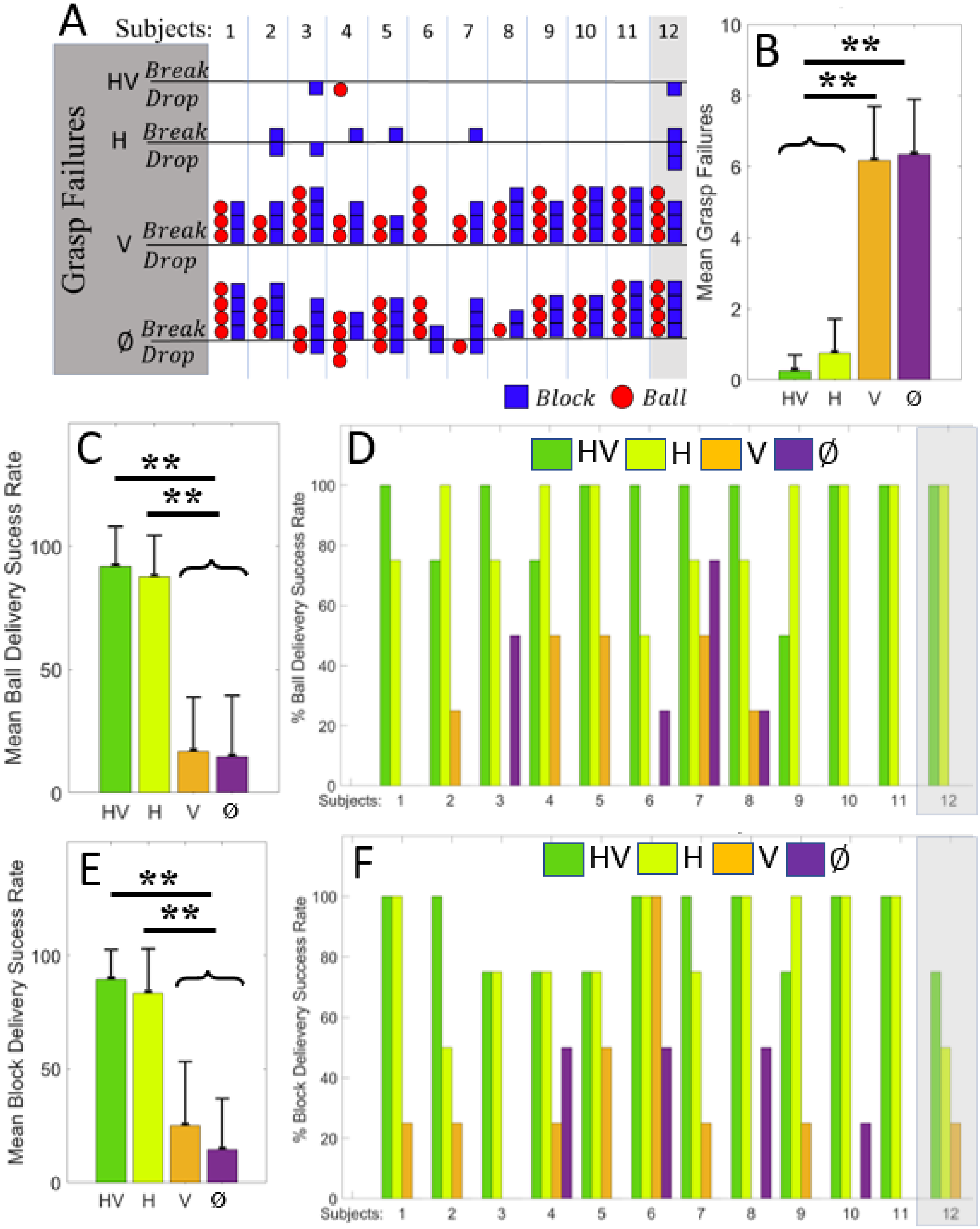

Fig. S6. Success and failure rates of the simultaneous object delivery task during the non-randomized experiments. (A) Number of break and drop failures that occurred with each object during sequential trials. Trials were conducted with Haptic and Vision (HV), with Haptic and no Vision (H), without Haptic but with Vision (V), without Haptic and without Vision (Ø). (B) Mean and standard deviation of failure data for all cases. (C,D) Success rates for non-randomized simultaneous control experiments with the ball and (E,F) block show a significant improvement with haptic feedback available. The gray highlight indicates the congenital limb-absent subject 12.

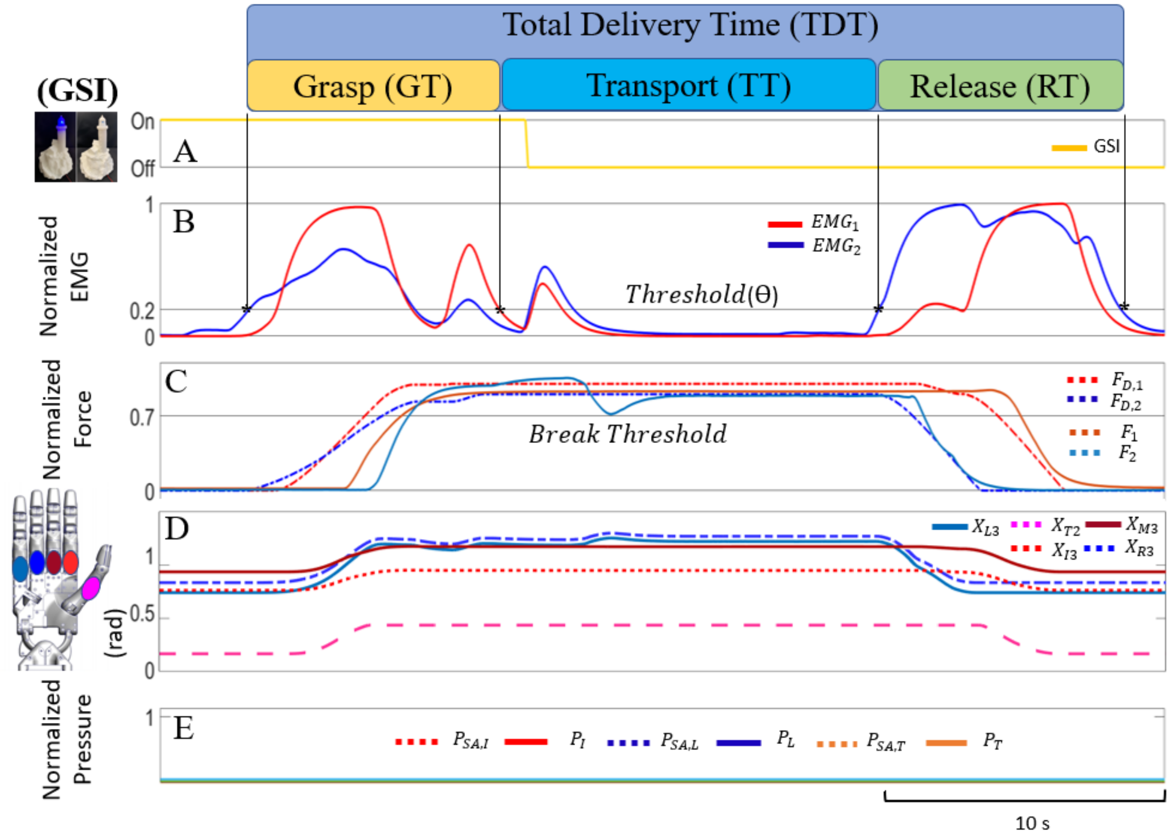

Fig. S7. Illustrative data showing both objects being 'broken' in the absence of haptic feedback. (A) Grip state indicator (GSI). (B) The subject increased both EMG_1 and EMG_2 simultaneously to close the hand and then subsequently increased the grip forces on both objects at the same time. Then a rapid co-contraction toggled the open/close state of the hand to the opening mode as it approached the delivery zones. Finally, the subject increased EMG_2 to deliver the block and then increased EMG_1 to drop the ball into the correct locations. (C) The desired forces passed the upper break threshold (0.7) which indicated that the objects were 'broken'. (D) Joint angles of the Shadow Hand increased and then decreased as the objects were grasped and released. (E) The pressures measured within the actuators of the soft robotic armband are all zero which means no haptic feedback was sent to the human subject.

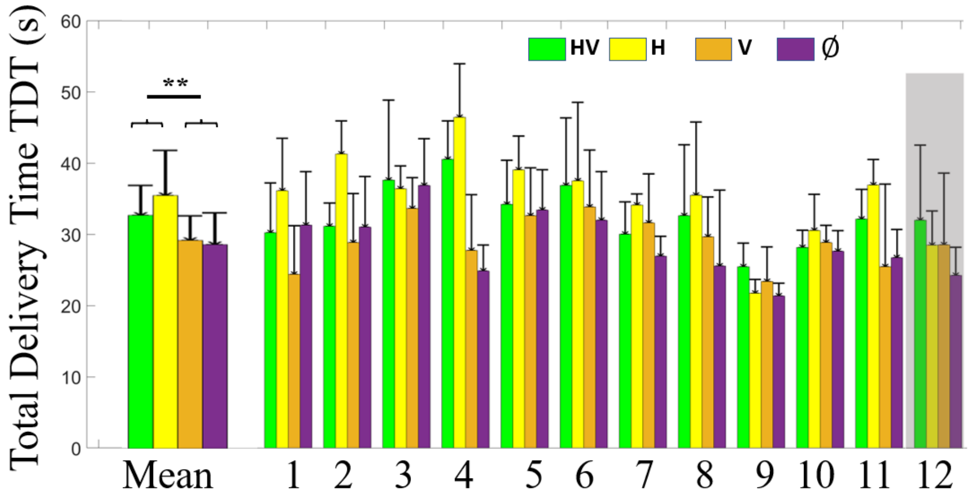

Fig. S8. Comparison of total delivery time required to grasp, transport, and deliver two objects simultaneously during the randomized experiments with the four possible combinations of independent variables: {1} haptic and visual feedback (HV), {2} only haptic feedback (H), {3} only visual feedback (V), and {4} neither haptic nor visual feedback (ϕ). The gray highlight indicates the congenital limb-absent subject 12.

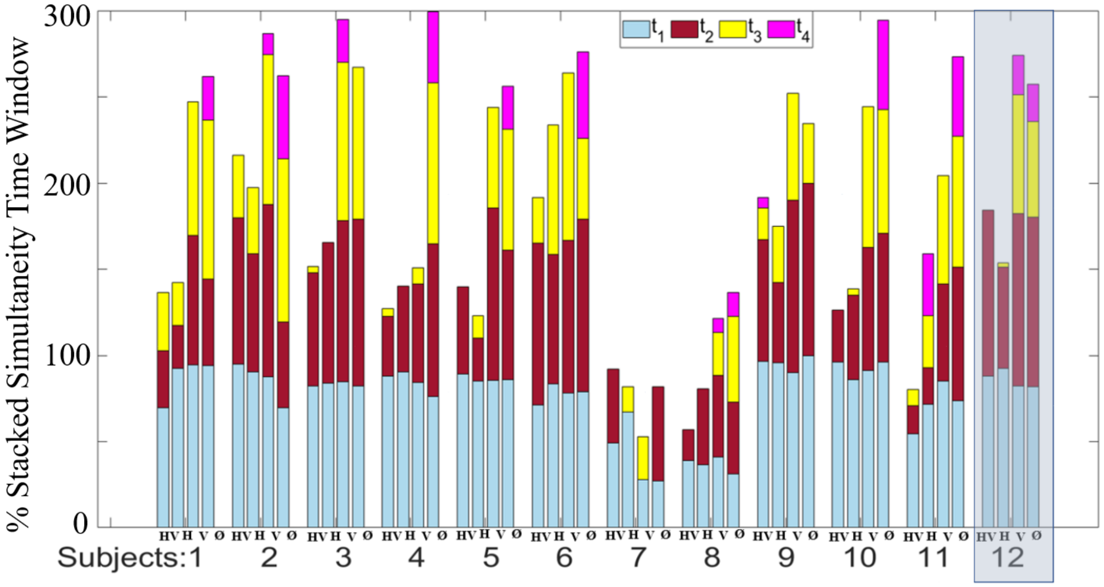

Fig. S9. Comparison of the simultaneity metric under the four-time windows, t_1, t_2, t_3, and t_4. See also eqn. 1 and Fig. 4. The grey highlighted subject 12 has a congenital limb absence.

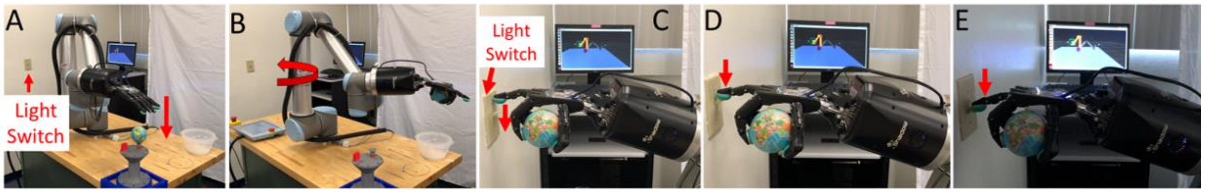

Fig. S10. The robotic arm performing two functions simultaneously: grasping and transporting a ball with a tripod grasp while flipping a light switch with the little finger. (A) Preparing to grasp the ball. (B) Shadow Hand grasped the ball and moved towards the light switch. (C-E) Hand in position to perform the second task: to flip the light switch to turn off the light while simultaneously maintaining a stable grasp on the ball. See also Movie S6.

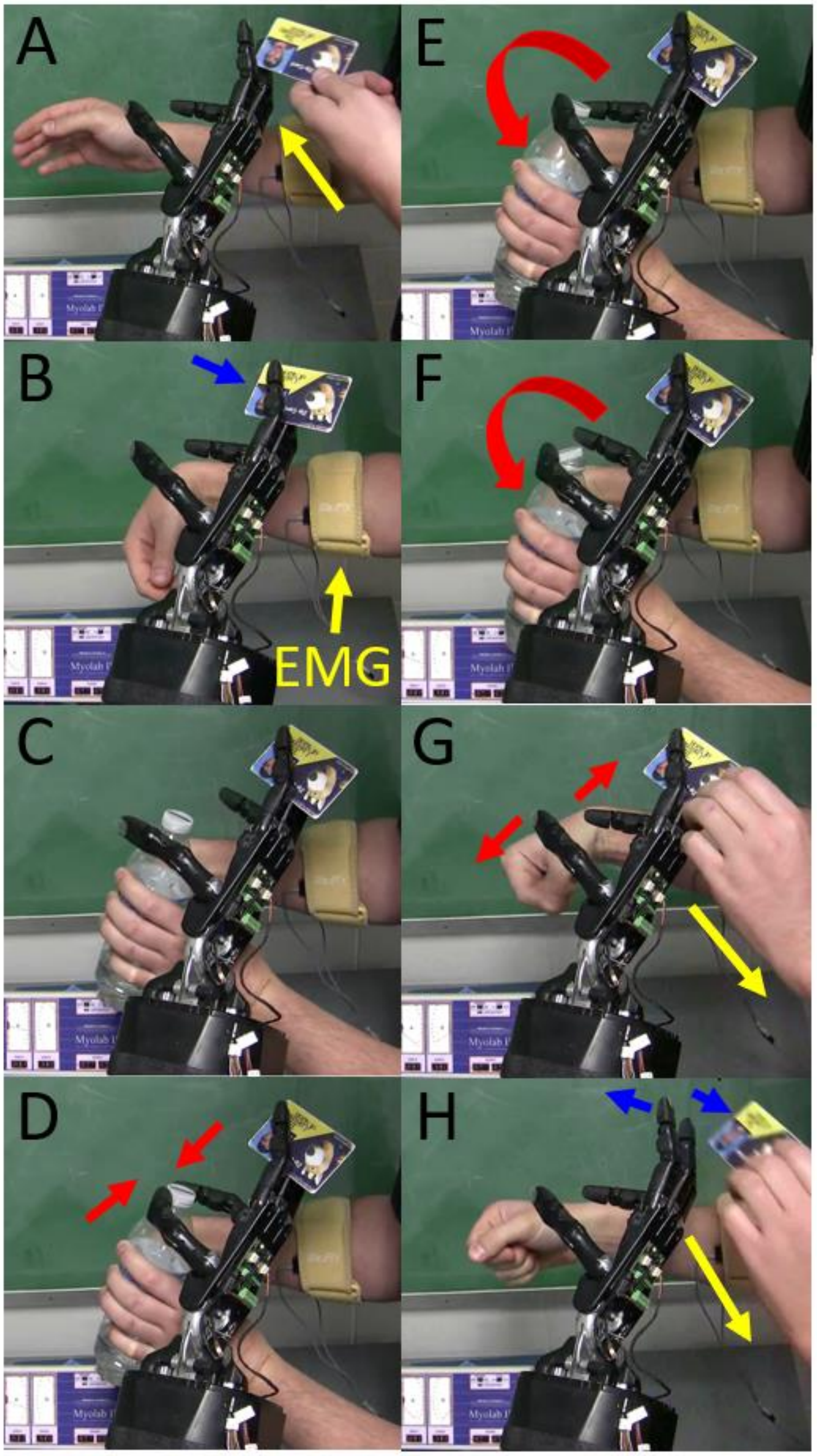

Fig. S11. Simultaneous EMG control of two different grip functions of the Shadow Hand. In this case, one EMG signal proportionally controlled the grip of the card between the index and middle fingers while the other EMG signal drove the unscrewing motion of the thumb and little finger (see also Movie S5).
